# Development and reliability testing of an audit toolbox for the assessment of the physical activity friendliness of urban and rural environments in Germany

**DOI:** 10.3389/fpubh.2023.1153088

**Published:** 2023-08-10

**Authors:** Christina Müller, Bruno Domokos, Tanja Amersbach, Eva-Maria Hausmayer, Christin Roßmann, Birgit Wallmann-Sperlich, Jens Bucksch

**Affiliations:** ^1^Institute of Sport Science, University of Wurzburg, Wurzburg, Germany; ^2^Department of Prevention and Health Promotion, Faculty of Natural and Sociological Sciences, Heidelberg University of Education, Heidelberg, Germany; ^3^Federal Centre for Health Education, Cologne, Germany

**Keywords:** built environment, physical activity, reliability, rural, urban, walkability

## Abstract

**Background:**

According to socio-ecological theories, physical activity behaviors are linked to the physical and social neighborhood environment. Reliable and contextually adapted instruments are needed to assess environmental characteristics related to physical activity. This work aims to develop an audit toolbox adapted to the German context, to urban and rural settings, for different population groups, and different types of physical activity; and to evaluate its inter-rater reliability.

**Methods:**

We conducted a systematic literature search to collect existing audit tools and to identify the latest evidence of environmental factors influencing physical activity in general, as well as in German populations. The results guided the construction of a category system for the toolbox. Items were assigned to the categories based on their relevance to physical activity and to the German context as well as their comprehensibility. We piloted the toolbox in different urban and rural areas (100 street segments, 15 parks, and 21 playgrounds) and calculated inter-rater reliability by Cohen's Kappa.

**Results:**

The audit toolbox comprises a basic streetscape audit with seven categories (land use and destinations, traffic safety, pedestrian infrastructure, cycling infrastructure, attractiveness, social environment, and subjective assessment), as well as supplementary tools for children and adolescents, seniors and people with impaired mobility, parks and public open spaces, playgrounds, and rural areas. 76 % of all included items had moderate, substantial, or almost perfect inter-rater reliability (κ > 0.4).

**Conclusions:**

The audit toolbox is an innovative and reliable instrument for the assessment of the physical activity friendliness of urban and rural environments in Germany.

## 1. Introduction

Physical activity, defined as “any bodily movement produced by skeletal muscles that requires energy expenditure,” has significant physical and mental health benefits (1). It reduces the risk of non-communicable diseases like cardiovascular diseases, type 2 diabetes, and cancer, as well as depressive symptoms and anxiety (2–7). However, physical inactivity is a major public health problem worldwide and also prevalent in all age groups in Germany (8–11).

To develop effective interventions, it is important to identify modifiable factors that positively or negatively influence physical activity behaviors. A growing number of studies have shown that beyond individual characteristics (e.g., intention), physical activity behaviors are linked to the physical (referring to the built and natural) and social neighborhood environment, as suggested by socio-ecological theories (12, 13). According to these theories, physical activity in different domains (e.g., occupational, leisure, transportation) is determined by different factors on different levels (i.e., intrapersonal, social, cultural, physical, information, and policy environment). The built environment, which can be defined as any human-made or human-modified features of the physical environment (e.g., buildings, transportation systems, design features, etc.), has gained attention from public health researchers, as interventions on this level potentially impact large proportions of the population (14, 15). A growing number of studies have confirmed that physical activity behaviors are linked to characteristics of the built environment, including accessibility, land use diversity, availability of public transport, aesthetics, infrastructure for walking and cycling, street connectivity, and traffic-related safety (16–20). Studies across different countries suggest that there are some internationally generalizable attributes of the built environment that are consistently associated with physical activity, but there might also be some country-specific influences (21–25). Furthermore, studies suggest that built environment influences differ across age groups. Hence, children, adolescents, adults, and older adults are usually examined as distinct groups, which is also necessary in terms of health reasons and determinants (18, 19, 26).

Children's and adolescents' physical activity is an important factor in their healthy growth and development (27). The World Health Organization (WHO) recommends a minimum of 60 min of moderate-to-vigorous-intensity physical activity (MVPA) per day for children and adolescents (1). In Germany, only small proportions of children and adolescents (22.4% of girls and 29.4% of boys between 3 and 17 years of age) achieve this recommendation, with prevalence continuously decreasing with age (9). Overall, boys accumulate more MVPA than girls (8, 9). Children's and adolescents' physical activity can be categorized into school-based activities (e.g., physical education), organized sports activities (e.g., in sports clubs), non-organized leisure activities (e.g., outdoor play), and active travel (e.g., walking or cycling to school) (28). The outdoor environment around children's and adolescent's homes and schools can contribute to their daily physical activity by offering opportunities for non-organized leisure activities and active travel (29). Outdoor time in general is positively associated with physical activity and negatively associated with sedentary behavior in children and adolescents (30). In addition, there is consistent evidence that active travel to and from school is positively associated with children's and adolescents' physical activity and contributes significantly to daily MVPA levels (31, 32). Environments that support walking and cycling as well as outdoor play can therefore be considered important for children and adolescents (33).

In German adults, 44.8% of women and 51.2% of men meet the WHO recommendation on aerobic physical activity (at least 150 min of moderate-intensity per week) (10). Active travel, including active commuting to work, is an important source of MVPA in adults (31, 34). Consequently, environments that support active travel, such as walking infrastructure (e.g., sidewalks), street connectivity, land-use mix, greater walkability, and proximity of destinations, are consistently associated with adults' physical activity (14).

In older adults, physical activity remains important, as it is known to be one of the key determinants of healthy aging. Physically active older adults have lower risks of falling, cognitive decline, dementia, and Alzheimer's disease (35). Studies have shown that retirement is associated with increases in leisure-time physical activity, (recreational) walking, and home-based activities and decreases in occupational physical activity, active travel, and total physical activity (36). In Germany, only 33.3% of women and 42.6% of men aged 65 and above achieve the WHO recommendations on aerobic physical activity of at least 150 min per week (10). Important domains of older adults' physical activity are home-based activities (e.g., household chores, gardening), leisure-activities (e.g., in sports clubs), and active travel (walking, cycling) (37). In the domain of active travel, walking is very important to maintain mobility with increasing age (38). According to a representative mobility survey, 43% of German adults aged 60 to 69 years, 47% of adults aged 70 to 79 years, and 44% of adults aged 80 years and older walk every day (38). Older adults are likely to spend much of their time in their neighborhoods and use them more intensively than younger adults (39). In addition, they may be more sensitive to physical barriers in the built environment because of age-related functional limitations (40). A systematic review of qualitative studies summarized that certain characteristics of the pedestrian infrastructure (e.g., sidewalk quality and maintenance, slopes, and curbs) and access to rest areas (i.e., benches and public washrooms) are important environmental factors for older adults, which is also supported by quantitative evidence (18, 41).

In recent years, different assessment instruments examining built environment characteristics related to physical activity have been developed, including audit tools (42, 43). Audit tools aim to measure the presence, quantity, and quality of environmental features by direct observation (42). They can be used by researchers as well as community stakeholders and have the potential to facilitate community assessments as part of a systematic planning process to improve environments for physical activity. Data is typically collected in-person using standardized paper forms or digital applications (42). To attain a high degree of independence from the observer, most tools have been tested for inter-rater reliability (42). Different reliable audit tools exist for different environmental contexts (e.g., street segments, parks, or public open spaces). Most of the tools have been developed in the United States, e.g., the Active Neighborhood Checklist (ANC) (44), the Microscale Audit of Pedestrian Streetscapes (MAPS) (45), the Pedestrian Environmental Data Scan (PEDS) (46), or the Rural Active Living Assessment (RALA) (47). Although most of the environmental characteristics assessed by these tools are supposed to have the same meaning in other Western high-income countries like Germany, there are also some culturally specific characteristics. For example, some differences between built environments in Europe and the United States have been described in the literature: European cities are often denser and more centralized than their US counterparts (48). US neighborhoods have historically been designed from an automobile perspective and are often hindering active travel, whereas European countries have been implementing car-restrictive policies and encouraging walking and cycling (e.g., through cycling infrastructure or traffic calming) since the 1970s (49, 50). In addition, a systematic review of the relationship between the physical environment and different domains of physical activity in Europe suggested that some environmental characteristics which are associated with physical activity in US adults might be less relevant for the physical activity of European adults (i.e., access to recreation facilities, aesthetics, and crime- and traffic-related safety) (51). Therefore, alternative measures may be more suitable in the European context (52). Audit tools developed and tested in other countries might not necessarily capture the most relevant characteristics of the physical activity-related built environment in Germany. Audit tools that are adapted to the local context are, on the one hand, less suitable to produce research data for cross-country comparisons, but on the other hand, they are more practicable for local stakeholders. Different audit tools have been tailored to European country-specific contexts, e.g., the Scottish Walkability Assessment Tool (SWAT) (53) or the Cyprus Neighborhood Observation Tool (Cy-NOTes) (54).

Only a few audit tools have been applied in Germany, with a focus on the urban pedestrian environment (55). However, a large proportion—between 15 and 60 percent of the German population lives in rural areas, depending on the applied definition of rurality (56). In Germany, rural areas are either defined as any municipality outside metropolitan areas (57), as any municipality with <5,000 inhabitants (58), or by an index of rurality combining lower density, a higher share of single-family homes, a higher share of agricultural land use and forests, lower demographic potential and lower accessibility of cities and towns (56). Apart from these defining factors, rural areas in Germany are diverse and differences between rural and urban areas in terms of lifestyles are decreasing (59). Although there is no significant difference in the total levels of physical activity of adults living in rural and those living in urban areas, adults in rural areas have lower levels of transport-related physical activity, which can be explained by longer travel distances to workplaces, shops, and other facilities and destinations (34, 60, 61). While older studies have found higher levels of physical activity in children and adolescents in rural compared to urban areas in Germany, a more recent one revealed stronger declines of total physical activity and outdoor play in rural areas, which may be a result of a lack of opportunities (62–65).

Internationally, only a few audit tools have been applied in rural areas [e.g., RALA, Inventories for Community Health Assessment in Rural Towns (ICHART) (47, 66)] and studies have suggested that some unique characteristics of rural areas (e.g., fewer destinations and longer distances) need special attention when assessing the built environment related to physical activity (47, 66, 67).

To the authors' knowledge, there is no comprehensive audit tool suitable to assess the built environment related to different types of physical activity in urban and rural German municipalities. Hence, we aimed (1) to develop an audit toolbox adapted to the German context, urban and rural settings, different population groups, and different types of physical activity, and (2) to evaluate its inter-rater reliability.

## 2. Materials and methods

**Figure 1:**
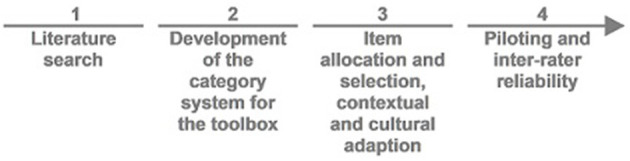
Development process of the KomBus-toolbox.

The KomBus audit toolbox (KomBus is a German acronym for “***Kom***munale ***B***ewegungsverhältnisse ***u***nter***s***uchen” = assess community environments for physical activity) was developed in four consecutive steps (Figure 1). In the first step, we conducted a systematic literature search to collect existing audit tools. In the second step, we created a category system for the toolbox building on the literature. In the third step, we assigned the content of the identified audit tools to the categories of the category system to receive an overview of relevant items. We then decided which items to include in the first draft of the audit toolbox. In the final fourth step, the first version of the audit toolbox was piloted and tested for inter-rater reliability.

### 2.1. Step 1: literature search

By reviewing existing audit instruments, we aimed to identify similarities and differences and derive constructs that can be reliably assessed by an audit tool. This approach has been chosen in the development of other audit tools (66, 68, 69). We identified existing audit tools for assessing community physical activity environments through a literature search in PubMed. The search strategy included keywords related to four different themes (see Supplementary material for the complete list of keywords):

- forms of physical activity (e.g., walk^*^, cycle^*^, active play, exercise)
- instruments designed to describe the environment (e.g., observational instrument, assessment tool, checklist)
- environmental attributes relevant to physical activity (e.g., walkability, bikeability, aesthetics, safety)
- study area and spaces (e.g., urban, rural, neighborhood, street^*^, open space^*^, green space^*^, playground)

In addition to the literature search in PubMed, two national databases (SPOLIT, LIVIVO) were searched with the same keywords in German. Detailed information was extracted from the articles on the names and types of the applied audit instruments as well as the geographical context (urban and/or rural context, continent, and country), the population group of interest, and relevant quality criteria (validity and reliability). During this process, bibliographical references of relevant articles were also screened.

### 2.2. Step 2: development of the category system for the toolbox

The categories for the toolbox were selected from existing categorizations of environmental characteristics assessed by audit tools (43, 70, 71) as well as the domains assessed by the tools identified in step 1 based on their relevance for physical activity and the availability of any evidence for their relevance in Germany. We inspected the results of 17 systematic reviews and one umbrella review on the relationship between characteristics of the built environment and physical activity in different population groups (see Supplementary Tables 2–5) (17–19, 51, 72–85). The selected categories and their evidence base are displayed in Table 1.

**Table 1:** Selection of categories.

**Category**	**Covered by existing audits**	**Relevance for physical activity**	**Relevance in Germany**
**Land use & destinations** - Primary land use - Services - Public places - Residential buildings - Presence of public transport - Frequency of public transport - Bicycle racks at transit stops	ANC, BiWET, BTG-COMP Street Segment Observation Form, CDC-HAN, CUBEST, CyNOTes, EAST-HK, EGA-Cycling, HEAT, iCHART, IMI, Instrument to assess health-affecting aspects of neighborhood in Tehran, MAPS, NAI, NALP, NBOT, NOC, OPECR, PHRESH, Physical Activity and Nutrition Features audit tool, PIN3, RALA, Rural Pedestrian Environmental Audit Tool, SPACES, SPACES for Alleys, SPOTLIGHT, St. Louis Analytic and Checklist Audit Tool, Street Design Environmental Audit, SWAN, SWAT, SWEAT-R, WASABE, WRATS	- Children's and adolescents unspecified PA (79) - Adults' and older adults' active travel and unspecified PA (18, 51, 81–83)	- Population density is positively associated with active travel in adults (34) - Residential density is associated with walking for transport in older adults (95) - Distance to destinations is associated with active travel (84, 95–97) - Having destinations like shops, healthcare, or recreational facilities within walking distance is important for older adults as well as families with children (98, 99) - Destinations related to sports or recreation are associated with PA in adolescents (84, 100, 101) - Availability/accessibility of public transport is important for older adults and associated with PA in adults (84, 98, 102–104) - The closer the bus stop, the higher the odds of older adults engaging in any walking for transport (95)
**Traffic safety** - Street type - Speed limit - Traffic volume - Parking - Safety measures	BiWET, BTG-COMP Street Segment Observation Form, CDC-HAN HEAT, EAST-HK, EP-NET, iCHART, IMI, Measure of environmental characteristics, NALP, OPECR, PEDS, PEQI, PHRESH, Physical Activity and Nutrition Features audit tool, PIN3, RALA, St. Louis Analytic and Checklist Audit Tool, Street Design Environmental Audit, SWAN, SWAT, SWEAT-R, WASABE, WEAT-D, WRATS	- Children's active travel and outdoor play/ activity (19, 72, 74, 78, 79) - Adults' leisure-time walking and cycling (51) - Older adults' walking and unspecified PA (18)	- Traffic safety is associated with older adults' walking for transport (95) - Older adults appreciate areas of reduced traffic and safe traffic conditions (103) - Parents prefer less traffic and less crossings on the way to school (99) - Traffic calming measures (e.g., speed limits) are a priority (105, 106) - Lack of safety, dangerous routes and too much traffic are barriers to children's active school transport (107, 108)
**Pedestrian environment** - Sidewalk type, continuity, demarcation, width, condition, material - Permanent obstacles - Temporary obstacles - Pedestrian signage - Slope - Crossing options/aids - Cross connections within segment and inter-segment	ANC, Assessment of the local outdoor environment for falling over, BiWET, BTG-COMP Street Segment Observation Form, CDC-HAN HEAT, CUBEST, CyNOTes, EAST-HK, EGA-Cycling, EP-NET, iCHART, IMI, IPSI, MAPS, MAUAP, Measure of environmental characteristics, NOC, NSAT, NWA, OPECR, OPERAT, PEDS, PEQI, PHRESH, Physical Activity and Nutrition Features audit tool, PIN3, RALA, Rural Pedestrian Environmental Audit Tool, Sidewalk Assessment Tool, SPACES, SPOTLIGHT, St. Louis Analytic and Checklist Audit Tool, Street Design Environmental Audit, SWAN, SWAT, SWEAT-R, WABSA, WASABE, WEAT-D	- Active travel in all age groups (17–19, 51, 74, 75, 78)	- Walkability is associated with unspecified PA and walking (84) - Walking infrastructure is positively associated with walking for transport in older adults (95) - Maintained sidewalks are associated with PA in adults in urban areas (104) - Lack of safe crossing option on major road is seen as a barrier to walking (106)
**Cycling environment** - Bicycle lane type, continuity, demarcation, width, condition, material - Permanent obstacles - Temporary obstacles - Cycling signage - Slope - Crossing options - Bicycle racks (types, number, occupancy) - Bicycle rental - Cross connections within segment and inter-segment	BiWET, CUBEST, CyNOTes, EGA-Cycling, EP-NET, iCHART, IMI, PHRESH, Physical Activity and Nutrition Features audit tool, SPACES, SPOTLIGHT, St. Louis Analytic and Checklist Audit Tool, Street Design Environmental Audit, SWAT, WABSA, WASABE	- Adults' active travel (51, 82, 85) - Children's, adolescents, and older adults' unspecified PA (17)	- Cycling infrastructure is positively associated with cycling in older adults (96) - Lack of cycling lanes are perceived as a barrier to cycling by parents and children (108, 109) - Lack of safe crossing option on major road is seen as a barrier to cycling (106)
**Attractiveness** - Building condition - Aesthetic buildings - Trees - Urban greenery - Aesthetic elements - Shade - Odors - Noise - Seating opportunities - Seating features - Toilets - Trash bins - Litter - Dog excrements - Weather obstructions	ANC, African American Health, BiWET, BTG-COMP Street Segment Observation Form, CDC-HAN HEAT, CUBEST, CyNOTes, EAST-HK, EGA-Cycling, EP-NET, iCHART, IMI, Instrument to assess health-affecting aspects of neighborhood in Tehran, MAPS, Measure of environmental characteristics, NAI, NBOT, NOC, NSAT, NWA, OPECR, OPERAT, PEDS, PEQI, PHRESH, PIN3, REAT, Rural Pedestrian Environmental Audit Tool, SPACES, SPACES for Alleys, SPOTLIGHT, St. Louis Analytic and Checklist Audit Tool, Street Design Environmental Audit, SWAN, SWAT, SWEAT-R, WalkBoston, WASABE, WEAT-D, WRATS	- Adults' cycling for transportation (82) - Older adults' unspecified PA (18) - Children's unspecified PA (incivilities/disorders) (79)	- Older adults prefer shaded footpaths/ sidewalks with benches/sitting facilities (103, 110) - Lack of sitting facilities and public toilets is a barrier to older adults' PA (111–113) - Lack of cleanliness is seen as a barrier (105) - Aesthetics of the built environment is associated with older adults' outdoor PA and walking for transport (95, 114)
**Social environment** - Number of people present - Physical activities - Age groups - Social interactions - Unpleasant people	CDC-HAN HEAT, CyNOTes, EAST-HK, iCHART, NAI, NBOT, NOC, PIN3, Rural Pedestrian Environmental Audit Tool, St. Louis Analytic and Checklist Audit Tool, SWAN, WASABE	- Children's and older adults' unspecified PA (18, 75)	- Item “I see many people being physically active” associated with PA in adults in urban areas (104) - Parents are concerned about the social environment of their children (e.g., drunk and scary people) (105)
**Subjective assessment** - Aesthetics - Pedestrian friendliness - Cycling friendliness - Sense of security - Quality of stay - Opportunities for social interaction	CSR, EP-NET, NBOT, NWA, PEDS, PEQI, RALA, SPACES, SWAN, SWAT	- Supposed to reflect the overall quality of the environment	
**Children & adolescents** - Playing on the street - Traffic calming measures (speed limit, built measures, signage) - Public places and destinations including accessibility	BTG-COMP Street Segment Observation Form, NWA, “Raum für Kinderspiel Wohnumfeldinventar”, Street Design Environmental Audit	- Less traffic and/or higher safety increases outdoor play/activity (19, 72) - Distance to relevant destinations (e.g., school) is associated with children's active travel (19, 75, 78)	- Lack of traffic safety is a reason for parents not to let their children play outside (105, 115) - Quality of the outdoor environment (e.g., no busy street, availability of playground) is associated with children's outdoor play time (116)
**Seniors & people with impaired mobility** - Barrier-free usage of ground - Slope eligibility using wheelchair or walker - Pedestrian lights including time of green phase - Curb cuts - Stairs/steps	Assessment of the local outdoor environment for falling over, CDC-HAN HEAT, MAUAP, OPECR, OPERAT, SWAN, SWEAT-R, WRATS	- Older adults do not like the presence of a steep gradient; when there are hills or stairs, they like the presence of handrails (41) - Older adults dislike cracked, uneven, steeply sloped, or high curbs that are impossible to negotiate with a walker (41) - Uneven or narrow sidewalks, rough pavement, absent curb cuts or those that are too high, poorly designed, or obstructed, are barriers for people using mobility assistive technology (117)	- Sidewalks that are too narrow and uneven are seen as a barrier (106) - Short light cycles at traffic lights can make senior citizens feel vulnerable, which prevents them from leaving their home and becoming active (103) - In addition to safety-features, barrier-free paths are rated as important by around 60% of all older adults (and more often by those with mobility impairments) (118)
**Parks & public open spaces** - Access and environment - Design, amenities, and activities - Condition, cleanliness, and safety - Social environment	BRAT-DO, BTG-COMP Park Observation Form, CPAT, C-POST, EARPS, NEST, NGST, PARK, Parks and Play Spaces Audit, POST, REDI, SAGE	- Attributes associated with park use and PA: variety of facilities (e.g., playgrounds), amenities (e.g., picnic tables), dog-specific equipment (for dog owners), shade, condition, accessibility, aesthetics, safety, and social environment (119) - Paths/trails and lighting promote park-based PA (120) - Presence of trails/walking paths, sport- and adventurous playgrounds are positively associated with public open space use by adolescents (76) - Lack of age-appropriate features is negatively associated with public open space use by adolescents (76)	- Natural areas should be designed attractively (105) - Children and adolescents appreciate diverse activity options, like basketball or soccer courts and outdoor fitness equipment (105)
**Playgrounds** - Access and environment - Design, amenities, and activities - Condition, cleanliness, and safety	Americas Playgrounds Safety Report Card, PARK, Parks and Play Spaces Audit, PSAT	- Large observational study in the U.S.: each additional play element was associated with about 50% more users and 50% more MVPA; playgrounds with on-site restrooms have more person-hours of use (121)	- Higher number of (active) children in playgrounds with more varied play facilities (122) - Parents prefer clean, safe, and attractive playgrounds (105, 106)
**Rural areas** - Settlement type - Distance to and accessibility of destinations - Special aspects	iCHART, RALA, Rural Pedestrian Environmental Audit Tool	- Lengthy travel distances, isolation, and lack of public transportation may be the largest barriers to PA in rural areas for children who rely on adults for transportation (123) - Environmental barriers to PA in rural areas include distance, safety of pedestrians among fast traffic and commercial trucks, and missing sidewalks (123)	- Distances are a barrier to active transport, especially, when there is no cycling infrastructure (34, 91, 109)

The literature search underlined the idea that a customization according to the investigator's intention (e.g., focusing on seniors or on rural settings) was useful. Therefore, the basic categories were complemented with additional categories sensitive to the peculiarities of specific contexts, settings, and population groups.

### 2.3. Step 3: item allocation and selection, contextual and cultural adaptation

After the definition of relevant categories and themes, possible items were extracted from the identified existing audit tools and assigned to them. By taking existing reliable and/or validated tools as a basis, we aimed to increase the quality of the KomBus toolbox. To identify the most applicable items for the KomBus toolbox, the following criteria were applied:

- relevance regarding physical activity (leisure activity, active transport) of different target groups (children and adolescents, adults, older adults) (assessment based on systematic reviews)
- relevance for the German context (assessment based on qualitative and quantitative studies from Germany, consideration of national traffic regulations and other country-specific definitions)
- comprehensibility, simplicity, and clarity.

For example, “types of residential buildings” is an item in many existing audit tools (e.g., 44, 53, 66) contributing to the description of the land-use mix and residential density, which are associated with physical activity in different population groups (18, 72, 79, 81, 82). A population-based German study has found a relationship between population density and active travel in adults (34). Therefore, the item “types of residential buildings” was considered to be relevant for the KomBus toolbox. We compared the operationalizations and response options in different audit tools and chose the most appropriate ones, considering the frequency of different residential buildings in German municipalities (see Table 2). Considering these criteria like in the example, the categories of the basic tool and the supplementary tools were filled with items. Eventually, these items were translated into German to generate a draft version of the toolbox ready for piloting.

**Table 2:** Selection of items (example for types of residential buildings).

**Active neighborhood checklist**	**EAST-HK**	**iCHART**	**MAPS global**	**St. Louis checklist audit tool**	**SWAT**	**KomBus basic tool**
**What types of residential uses are present?** Select all that apply.	**What types of residential uses do you see?** Check all that apply.	**What are the types of housing in this community?**	**What types of residential uses?** Check all that apply	**Types of residential destinations**	**Residential buildings**	**What types of residential buildings are present?**
□ None	□ Single-family homes	□ Abandoned homes	□ Single family houses	□ Single-family home	□ Detached houses	□ Single-family house (detached)
□ Abandoned homes	□ Multi-family homes	□ Rentals (e.g., apartments, duplexes)	□ Multi-unit homes (duplex, 4-plex, row house)	□ Two-, three-, four-, five-, or six-family home	□ Semi-detached houses	□ Semi-detached/terrace house
□ Single family homes	□ 4-6 floors apartment blocks	□ Single-family homes	□ Apartment or condominiums	□ Apartment building/ complex or condominium	□ Terrace houses	□ Apartment building (up to 4 units)
□ Multi-unit homes (2-4 units)	□ 7-12 floors apartment blocks	□ Mobile homes/ trailers	□ Apartments above street retail	□ Apartment over retail in multi-story building	□ Flats/ tenements	□ Apartment building (> 4 units)
□ Apartments or condominiums (>4 units, 1-4 stories)	□ 13-20 floors apartment blocks	□ Other	□ None of the above	□ Mobile home or trailer	□ High-rise flats	□ Other
□ Apartments or condominiums (>4 stories)	□ Over 20 floors apartment blocks			□ Mobile home or trailer park/ community	□ Flats over retail	□ None
□ Apartment over retail				□ Other	□ Other	
□ Other (retirement home, mobile home, dorms)						

### 2.4. Step 4: piloting and inter-rater reliability

The initial draft of the KomBus-toolbox was field-tested with a focus on feasibility and comprehensibility. Three researchers carried out primal field tests with the toolbox. Besides, a manual containing further explications, definitions, and reference photos was created to assist auditors.

Additional feedback on the toolbox was obtained from student field testers from two different universities. Incorporating feedback from these initial field tests, the tool was revised multiple times before piloting for inter-rater reliability.

In July and August 2021, 100 street segments were assessed using the basic tool and the supplementary tools for *children and adolescents* and *seniors and people with impaired mobility*. We determined the minimum number of street segments required for calculating the inter-rater reliability of the basic tool following the recommendations by Bujang and Baharum (86). The mean number of response options (two), the minimum value for the desired kappa coefficient (0.4), the desired power (80.0%), the specified alpha-value (0.05), as well as the assumption that the proportions in each response option are not proportional to each other, determined a minimum sample of 94 street segments (86). Accordingly, we set the sample size at 100 street segments. In addition, 15 parks and 21 playgrounds were assessed using the supplementary tools for *parks and public open spaces* and *playgrounds*. Two different auditors independently assessed each street segment, park, and playground. Cities and communities of different sizes were included: Frankfurt on the Main (population: 764,104; density: 3,100/km^2^), Wurzburg (population: 126,954; density: 1,450/km^2^), Schweinfurt (population: 53,319; density: 1,500/km^2^), Karlstadt on the Main (population: 14,930; density: 152/km^2^), Hoechberg (population: 9,501; density: 1250/km^2^) and two communities in the rural district Rhoen-Grabfeld (Wuelfershausen an der Saale; population: 1,501; density: 83/km^2^; Saal an der Saale; population: 1,517; density: 70/km^2^) (87).

Data was processed with SPSS Statistics (see Supplementary material for the datasets). Inter-rater reliability was calculated for all categorical variables with sufficient variability using Cohen's Kappa statistic (κ), defined as:


$$\begin{array}{l} \kappa =\frac{p_{0}-p_{e}}{1-p_{e}} \end{array}$$

where p_0_ is the relative observed agreement between raters and pe is the hypothetical probability of chance agreement. Kappa values were classified as suggested by Landis and Koch, with almost perfect defined as κ ≥ 0.81, substantial defined as 0.6 < κ ≤ 0.8, moderate defined as 0.4 < κ ≤ 0.6, fair defined as 0.2 < κ ≤ 0.4, slight defined as 0.0 < κ ≤ 0.2, and poor defined as κ < 0.0 (88).

## 3. Results

### 3.1. Step 1: literature search

The PubMed search identified 692 articles that were title-screened for relevance. After excluding 440 articles, 252 articles remained for the full-text screening process. Information on the applied audit tools (name and type), the geographical context (urban and/or rural context, continent, and country), the population group of interest, and relevant quality criteria (validity and reliability) was retrieved. In sum, 86 audit instruments were identified across the 252 articles and their references. Additionally, four German tools were identified. The extracted information on the identified tools is summarized in Supplementary Table 1.

### 3.2. Step 2: category system of the toolbox: “basic tool”and “supplementary tools”

Seven categories were defined as basic categories irrespective of the population group and rurality: (A) *land use* (B) *traffic safety*, (C) *pedestrian environment*, (D) *cycling environment*, (E) *attractiveness*,(F) *social environment*, and (G) *subjective assessment*. Deriving from the literature search results, the main population groups of interest not fully covered in the basic tool were *children and adolescents* as well as *seniors and people with impaired mobility*. Hence, we decided to develop two supplementary tools with specific categories incorporating items of high relevance for those groups. In the case of children and adolescents, the categories were *traffic calming* and *public places and destinations*. For seniors and people with impaired mobility, the category *demands of people with impaired mobility* was added. Two independent supplementary tools were developed for *parks and public open spaces* and *playgrounds*. The two supplementary tools were constructed similarly with both tools containing the categories A) *access and environment, B) design, amenities, and activities* as well as C) *condition, cleanliness, and safety*. The parks and public open spaces tool was supplemented by the category D) *social environment*.

Although the definition of rurality is complex and adequate measures are debatable (89), rural settings are often characterized by longer travel distances to destinations and a lower land use mix. Therefore, it was considered useful to develop a supplementary tool for *rural areas*, which looks beyond the streetscape of a particular segment, considering active travel options to destinations in a travel radius of 20 km.

### 3.3. Step 3: selected and adapted themes for the “basic tool” and for the “supplementary tools”

The categories and themes of the final version of the “Basic Tool” are displayed in Table 3.

**Table 3:** Categories and themes of the KomBus basic tool.

**A: land use and destinations**	**B: traffic safety**	**C: pedestrian environment**	**D: cycling environment**	**E: attractiveness**	**F: social environment**	**G: subjective assessment**
*7 themes*: Primary land use Services Public places Residential buildings Presence of public transport Frequency of public transport Bicycle racks at transit stops	*5 themes*: Street type Speed limit Traffic volume Parking Safety measures	*14 themes:* Sidewalk type Sidewalk continuity Sidewalk demarcation Sidewalk width Sidewalk condition Sidewalk material Permanent obstacles Temporary obstacles Pedestrian signage Slope Crossing options Crossing aids Cross connections (within-segment) Cross connections (inter-segment)	*15 themes:* Bicycle lane type Bicycle lane continuity Bicycle lane demarcation Bicycle lane width Bicycle lane condition Bicycle lane material Permanent obstacles Temporary obstacles Cycling signage Slope Crossing options Bicycle racks (types, number, occupancy) Bicycle rental Cross connections (within-segment) Cross connections (inter-segment)	*15 themes:* Building condition Aesthetic buildings Trees Urban greenery Aesthetic elements Shade Odors Noise Seating opportunities Seating features Toilets Trash bins Litter Dog excrements Weather obstructions	*5 themes:* Number of people present Physical activities Age groups Social interactions Unpleasant people	*6 themes:* Aesthetics Pedestrian friendliness Cycling friendliness Sense of security Quality of stay Opportunities for social interaction

The supplementary tools are listed in Table 4. The supplements were designed to be used optionally, depending on the investigators' goal.

**Table 4:** Categories and themes of the KomBus supplementary tools.

**H: children and adolescents**	**I: seniors and people with impaired mobility**	**J: parks and public open spaces**		**K: playgrounds**		**L: rural areas**
*3 themes:* Playing on the street Traffic calming measures (speed limit, built measures, signage) Public places and destinations including accessibility	*5 themes:* Barrier-free usage of ground Slope eligibility using wheelchair or walker Pedestrian lights including time of green phase Curb cuts Stairs/steps	*9 themes— access and environment:* Signposts Opening hours Entrance (quantity) Barrier-free entrance Arrival options and parking Information signs Environment Possible uses Overall use *5 themes— condition, cleanliness, and safety:* Park size Fencing Path types Upkeep Inconveniences	*7 themes— design, amenities, and activities:* Physical activities and sports Seating areas Lighting Shade Amenities and condition Noise Dog regulations *4 themes—social environment:* Age groups Social interactions Physical activities Unpleasant people	*2 themes— access and environment:* Arrival options and parking Information signs *8 themes—design, amenities, and activities:* Play equipment Creative play Age groups Separation of age groups Size Physical activity options environment Shade Amenities	*5 themes— condition, cleanliness, and safety:* Defective equipment Fencing Inconveniences Upkeep Overall impression	*4 themes— settlement type:* Type of settlement investigated Distance and travel options to upper center Distance and travel options to middle center Distance and travel options to lower center *2 themes—special aspects:* Industrial vehicles Livestock 22 themes —destinations and accessibility^*^

^*^Distance and travel options (walking, cycling, public transport) to the following destinations: grocery store/supermarket, childcare center/kindergarten, elementary school, comprehensive school, secondary school—“Mittel-/Hauptschule”, secondary school—“Realschule”, secondary school—“Gymnasium”, vocational school, other school, pharmacy, general practitioner, medical specialist, dentist, park/public green space, playground, sports facilities, swimming pool/lake, gym, hiking/cycling trail, public meeting place, and other destinations (2x).

### 3.4. Step 4: piloting and inter-rater reliability

76 % of all included items had moderate, substantial, or almost perfect inter-rater reliability (Basic Tool: 71%, supplementary tool *children and adolescents*: 76%, supplementary tool *seniors and people with impaired mobility*: 100%, supplementary tool *parks and public open spaces*: 78%, supplementary tool *playgrounds*: 85%). Table 5 summarizes the results by category.

**Table 5:** Aggregated results for inter-rater reliability.

	**No. of items**	**Inter-rater reliability (Cohen's Kappa** ^**b**^ **)**								**% agree-ment, range**
		**Included items** ^a^	**Almost perfect**	**Substantial**	**Moderate**	**Fair**	**Slight**	**Poor**	**% moderate, substantial, or almost perfect**	
Land use and destinations	19	18	3	11	3	1	0	0	94%	83–100%
Traffic safety	13	13	1	5	5	0	1	1	85%	69–100%
Pedestrian environment	65	58	10	12	14	17	1	4	62%	68–100%
Cycling environment	67	46	7	18	12	5	3	1	80%	64–100%
Attractiveness	26	20	5	3	6	5	0	1	70%	50–100%
Social environment	14	13	2	4	1	5	1	0	54%	58–99%
Subjective assessment	6	6	0	0	1	1	4	0	17%	32–65%
Children & adolescents	95	34	6	13	7	3	1	4	76%	0−100%
Seniors and people with impaired mobility	15	8	2	3	3	0	0	0	100%	80–100%
Parks and public open spaces	110	69	20	18	16	6	5	4	78%	36–100%
Playgrounds	65	55	27	17	3	6	0	2	85%	65–100%
All parts	495	340	83	104	71	49	16	17	76%	

^a^Items with no variability were excluded from the analysis.

^b^Almost perfect is defined as κ ≥ 0.81, Substantial is defined as 0.6 < κ ≤ 0.8, Moderate is defined as 0.4 < κ ≤ 0.6, Fair is defined as 0.2 < κ ≤ 0.4, Slight is defined as 0.0 < κ ≤ 0.2, Poor is defined as κ < 0.0 (88).

## 4. Discussion

In this study, we developed and tested an audit toolbox adapted to the German context, urban and rural settings, different population groups, and different types of physical activity. The toolbox consists of a basic tool with seven categories (land use and destinations, traffic safety, pedestrian environment, cycling environment, attractiveness, social environment, and subjective assessment) and five supplementary tools (children and adolescents, seniors and people with impaired mobility, parks and public open spaces, playgrounds, and rural areas). Across all parts of the toolbox, most of the items demonstrated at least moderate inter-rater reliability (κ > 0.4). Of the seven categories of the basic tool, the category *land use and destinations* showed the highest reliability. Of the supplementary tools, the tool *seniors and people with impaired mobility* showed the highest reliability. The category *subjective assessment* demonstrated the lowest inter-rater reliability, followed by *social environment* and *pedestrian environment*.

To our knowledge, KomBus is the first comprehensive audit toolbox composed of different parts that can be used depending on the context and population group of interest. Compared to other audit instruments [e.g., SWAT, RALA, MAPS (45, 47, 53)], KomBus equally considers the needs of pedestrians and cyclists, taking into account the importance of cycling in Germany. Aspects related to safety from crime, like surveillance or street level windows, were considered less relevant in Germany and were thus not included. Another difference is the integration of open fields for the auditor to note any present destinations, as compared to lists of destinations found in other tools. This is supposed to facilitate the application of the audit in general and particularly in rural areas, where fewer destinations are present, and allows for a more precise description of the present destination.

Since limited access to destinations like schools, shops, recreation facilities, or healthcare services has been described as a barrier to active travel in rural areas (67, 90, 91), we developed a supplementary tool for rural communities to assess active travel options to destinations in a travel radius of 20 km. This supplementary tool is similar to the RALA townwide assessment (47), but allows for a more precise analysis of different travel options (walking, cycling, and public transport), potential barriers (e.g., lack of walking or cycling infrastructure, frequency of public transport), and a greater variety of destinations (e.g., pharmacy or supermarket).

Concerning inter-rater reliability, KomBus is comparable to other audits (42, 46, 92). Similar to other instruments, there were some items with relatively low kappa values. However, some of these can be explained by other reasons than actual low reliability. In some cases, little variation across segments led to low kappa values despite high observer agreement. This applied to most items of the category *pedestrian environment* with lower inter-rater reliability; e.g., the item “street furniture as permanent obstacle for pedestrians” had an observer agreement of 96 %, but a high prevalence of the “no”-category, which led to a kappa value defining poor inter-rater reliability (93). Other items with lower inter-rater reliability were subject to continuous change, e.g., the number of people present, and changes may have occurred between the assessments of the first and the second auditor. The low inter-rater reliability in the subjective assessment had been anticipated, given the subjective nature of this category.

Based on the results of the reliability testing and the identified reasons for lower kappa statistics, we decided which items should be excluded from the toolbox (items not prevalent in any of the 100 street segments, e.g., gravel as material of sidewalks) and which items required further explanation or clarification (e.g., condition of sidewalks). Despite their relatively low reliability, we decided not to exclude items of the subjective assessment and social environment, as these categories may be of additional value for community stakeholders applying the toolbox in their community.

Recent research has pointed out the potential of virtual audits using Google Street View as reliable, cost-effective, and time-effective alternatives to field audits (94). However, this is currently no viable option in Germany, as coverage is limited, especially outside metropolitan areas. Besides, field audits have some advantages compared to virtual audits: Field audits are more suitable to assess features susceptible to temporal variability (e.g., litter or temporary obstructions), as well as sensory impressions like noise or odors (94). In addition, we advocate for the exposition of the auditor in the real setting as it facilitates the deduction of needs for changes in the environment. The main strength of a field audit is to provide an extensive and accurate description of environmental attributes directly or indirectly influencing physical activity in different communal settings.

Using audit tools to quantify the physical activity friendliness of communities is not an easy process. The application of sum scores used in some other audits (e.g., MAPS), that aim to evaluate the quality of environments in an attempt to quantify favorable and unfavorable factors, was regarded as not expedient for this purpose. This would undermine the fact that the evaluation of attributes being either favorable or unfavorable to physical activity depends on the perspective of the user (e.g., cycling lanes vs. play roads). In place of sum scores, KomBus was complemented with a manual providing interpretation aids. The aids include descriptions of each component, information on evidence of promoting factors of physical activity related to settings and target groups, as well as questions that may help to contemplate measures for improvement.

We would like to mention some limitations. The toolbox was developed and tested by a team of experienced researchers through an extensive review of the literature. However, community stakeholders have not been involved in the development and testing of the toolbox. Although we tested the toolbox in a variety of neighborhoods in cities and villages of different sizes, some of the items lacked variability and could not be assessed for inter-rater reliability. The focus of the pilot test was the inter-rater reliability of the toolbox. To improve its usability for research purposes, future research should take an effort in assessing validity measures such as the internal consistency and the discriminant capacity between categories. Further research is also necessary to test whether levels of physical activity are higher in areas with more favorable activity-friendly environments assessed with the toolbox. This was not part of the current study. Besides, we recommend testing the toolbox in other parts of Germany or in other European countries. Another suggestion for further development would be to test the tool with urban planners, architects, or professionals working in the field of health promotion.

The toolbox is available online as a part of the digital planning tool for physical activity-friendly communities (https://www.aelter-werden-in-balance.de/impulsgeber-bewegungsfoerderung/) recently launched by the German Federal Center for Health Education (BZgA).

## 5. Conclusions

This paper presents the literature-based development and reliability-testing of the German audit toolbox KomBus considering characteristics of the built environment that are associated with physical activity in different population groups. The toolbox demonstrated moderate to good inter-rater reliability and can be recommended for use by researchers and community stakeholders in German urban and rural areas.

## Data availability statement

The original contributions presented in the study are included in the article/Supplementary material, further inquiries can be directed to the corresponding author.

## Author contributions

CM, BD, BW-S, and JB contributed to the research proposal and design. CM, BD, and TA conducted the literature search and developed the audit toolbox with continuous feedback from BW-S, JB, and CR. CM, BD, and TA conducted the field tests. CM, BD, and E-MH participated in data management. CM and E-MH conducted the reliability analyses. CM and BD drafted the manuscript. TA, E-MH, CR, BW-S, and JB edited the manuscript. All authors read and approved the final manuscript.
