# Supplementary material for: Development and reliability testing of an audit toolbox for the assessment of the physical activity friendliness of urban and rural environments in Germany
Source: Front Public Health. 2023 Aug 10;11:1153088. doi: 10.3389/fpubh.2023.1153088 (PMC10449332; doi:10.3389/fpubh.2023.1153088)
Supplement: Supplementary file 1 [file Table_1.DOCX]

Supplementary Material

Development and reliability testing of an audit toolbox for the assessment of the physical activity friendliness of urban and rural environments in Germany

Christina Müller*, Bruno Domokos, Tanja Amersbach, Eva-Maria Hausmayer, Christin Roßmann, Birgit Wallmann-Sperlich, Jens Bucksch

*** Correspondence:** Christina Müller: christina.mueller@stud-mail.uni-wuerzburg.de

# Supplementary Data

**Search-terms for the identification of existing audit tools**

"physical activity"[TIAB] OR "physical activities"[TIAB] OR "physical inactivity"[TIAB] OR "mobility"[TIAB] OR "walk*"[TIAB] OR "cycle"[TIAB] OR "cycling"[TIAB] OR "biking"[TIAB] OR “bicycle”[TIAB] OR "transport*"[TIAB] OR "commute"[TIAB] OR "commuting"[TIAB] OR “travel”[TIAB] OR “pedestrian”[TIAB] OR “active play”[TIAB] OR “sport*”[TIAB] OR “exercise”[TIAB]

AND

“audit*”[TIAB] OR “observational instrument*”[TIAB] OR "observation instrument*"[TIAB] OR “observational measure*”[TIAB] OR "assessment tool*"[TIAB] OR "checklist*"[TIAB]

AND

"environment*"[TIAB] OR “physical attributes”[TIAB] OR “physical characteristics”[TIAB] OR "socio-ecological"[TIAB] OR "traffic"[TIAB] OR "facilities"[TIAB] OR "walkability"[TIAB] OR “bikeability”[TIAB] OR “aesthetics”[TIAB] OR “safety”[TIAB]

AND

"neighbourhood*"[TIAB] OR "neighborhood*"[TIAB] OR "community"[TIAB] OR "communities"[TIAB] OR "community-based"[TIAB] OR "park*"[TIAB] OR "urban"[TIAB] OR "rural"[TIAB] OR “greenspace*”[TIAB] OR “open space*”[TIAB] OR “street*”[TIAB] OR “public space*”[TIAB] OR “playground*”[TIAB] OR “path*”[TIAB] OR “trail*”[TIAB] OR “recreation area*”[TIAB]

# Supplementary Figures and Tables

Supplementary table 1: Identified audit instruments

| **No.** | **Name of the audit** | **Type** | **Urban/ Rural** | **Population group** | **Continent, country** | **Quality criteria** | **Ref.** |
| --- | --- | --- | --- | --- | --- | --- | --- |
| 1 | Active Neighborhood Checklist | Community/ Streetscape | Unspecific | Unspecific | North America, USA | **Inter-rater reliability**  Mean observed agreement: 0.87 (range 0.61-1.00).  Mean Cohen‘s kappa: 0.68 (range: 0.21-1.00 | (1) |
| 2 | African American Health | Community/ Streetscape | Urban | Seniors | North America, USA | **Discriminant and concurrent validity**  Strong and consistent differences in ratings between the inner city and suburb neighborhoods  **Convergent validity**  Association between overall scale and global neighborhood rating by residents | (2) |
| 3 | Assessment of the local outdoor environment for falling over | Community/ Streetscape | Urban | Seniors | Europe, UK | None reported | (3) |
| 4 | Bikeability and Walkability Evaluation Table (BiWET) | Community/ Streetscape | Urban | Unspecific | Europe, Austria | **Intra-rater reliability**  Mean observed agreement: 0.95 (range 0.89-0.98)  **Inter-rater reliability**  Mean observed agreement: 0.89 (range 0.79-0.97) | (4) |
| 5 | Bridging the Gap Community Obesity Measures Project (BTG-COMP) - Street Segment Observation Form | Community/ Streetscape | Urban | Children/ Adolescents | North America, USA | **Inter-rater reliability**  All items included in the index with almost perfect or substantial agreement (ICCs 0.61-1.00) or >90% observed agreement | (5) |
| 6 | CDC-Healthy Aging Research Network Environmental Audit Tool (CDC-HAN HEAT) | Community/ Streetscape | Urban | Seniors | North America, USA | **Inter-rater reliability**  Cohen’s kappa ≥ 0.4 | (6, 7) |
| 7 | Children’s Environmental Health Initiative - Community Assessment Project (CEHIs CAP) | Community/ Streetscape | Urban | Unspecific | North America, USA | **Inter-rater reliability**  Mean observed agreement: 0.95; agreement across all items > 0.70 | (8) |
| 8 | Checklist for Pedestrians’ Crossing areas | Community/ Streetscape | Urban | Unspecific | North America, Mexico | None reported | (9) |
| 9 | Community Health Environment Scan Survey (CHESS) | Community/ Streetscape | Urban | Unspecific | North America, Mexico | **Inter-rater reliability**  Consistently high agreement for the main variables (kappa and AC1 close to 1.0) | (10) |
| 10 | China Urban Built Environment Scan Tool (CUBEST) | Community/ Streetscape | Urban | Unspecific | Asia, China | **Intra-rater reliability**  ICCs for dimensions between 0.360 and 0.758  **Inter-rater reliability**  ICCs for dimensions between 0.766 and 0.912  **Construct validity**  Almost perfect matching between 6 components and the corresponding items | (11) |
| 11 | Community Street Review (CSR) | Community/ Streetscape | Urban | Unspecific | New Zealand | None reported | (12) |
| 12 | Cyprus Neighbourhood Observation Tool for auditing community environment (CY-NOTes) | Community/ Streetscape | Urban | Unspecific | Europe, Cyprus | **Content validity**  assessed by reviewing Facebook posts related to the neighborhood environment | (13) |
| 13 | Environment in Asia Scan Tool – Hong Kong (EAST-HK) | Community/ Streetscape | Urban | Unspecific | Asia, Hong Kong | **Intra-rater reliability**  59 items with substantial to perfect reliability  **Inter-rater reliability**  46 items with substantial to perfect reliability | (14) |
| 14 | Environmental Profile of a Community’s Health- Photos-Neighbourhood Evaluation Tool (EP-NET / EPOCH I) | Community/ Streetscape | Unspecific | Unspecific | Unspecific, Brazil, Canada, China, Colombia, India | **Inter-rater reliability**  77% (46 of 60) items had an ICC of ≥0.75, 17% (10 of 60) had an ICC between 0.60 and 0.74, 0% had an ICC between 0.4 and 0.59 and 5% (3 of 60) items had an ICC ≤0.40 | (15) |
| 15 | Inventories for Community Health Assessment in Rural Towns (ICHART) | Community/ Streetscape | Rural | Unspecific | North America, USA | **Intra-rater reliability**  Mean Cohen’s kappa: 0.72.  **Inter-rater reliability**  Mean observed agreement: 0.84 | (16) |
| 16 | Instrument to assess health-affecting aspects of neighborhood in Tehran | Community/ Streetscape | Urban | Unspecific | Asia, Iran | **Inter-rater reliability**  Median kappa of the final checklist: 0.78 (range 0.63-1.00)  **Content validity**  Confirmed by experts | (17) |
| 17 | Inventory for Pedestrian Safety Infrastructure (IPSI) | Community/ Streetscape | Urban | Unspecific | North America, USA | **Inter-rater reliability**  Cohen’s kappa between 0.05 and 1.0  ICCs between 0.192 and 1.0  **Internal consistency**  Intersection scale: Cronbach’s Alpha 0.86  Roadway scale: Cronbach’s Alpha 0.60 | (18) |
| 18 | Irvine Minnesota Inventory (IMI) | Community/ Streetscape | Urban | Unspecific | North America, USA | **Inter-rater reliability**  68% of the items had >70% agreement among three observers | (19) |
| 19 | Microscale Audit of Pedestrian Streetscapes (MAPS) / Full / Abbreviated / Mini / Global / Global-SN | Community/ Streetscape | Urban | Unspecific | North America, USA | MAPS Full  **Inter-rater reliability**  84.9% of items with moderate to excellent reliability (ICC or Cohen’s Kappa)  MAPS Abbreviated  **Validity**  Destinations and land use, streetscape and walking path characteristics, and overall total scores are related to active transport in all age groups.  MAPS Mini  **Validity**  Total scores are related to active transportation in all age groups.  MAPS Global  **Inter-rater reliability**  Median ICC: 0.92 (range 0.50-1.0)  MAPS Global-SN  **Inter-rater reliability**  ICCs between 0.60 and 0.99 | (20–24) |
| 20 | Measure of Accessibility to Urban Infrastructures for Adults with Physical Disabilities (MAUAP) | Community/ Streetscape | Urban | People with disabilities | North America, Canada | **Inter-rater reliability**  Gwet’s AC1 between 0.63 and 1.00  Observed agreement between 0.804 and 1.00 | (25) |
| 21 | Measure of environmental characteristics | Community/ Streetscape | Urban | Unspecific | North America, USA | **Inter-rater reliability**  ICC > 0.85 for all measures | (26) |
| 22 | Measuring Urban Design Qualities | Community/ Streetscape | Urban | Unspecific | North America, USA | **Inter-rater reliability**  ICCs between 0.344 and 0.584 | (27) |
| 23 | Neighbourhood Design Characteristics Checklist (NeDeCC) | Community/ Streetscape | Unspecific | Seniors | Europe, England | **Inter-rater reliability**  Kappa ≥ 0.6 for all categorical items | (28) |
| 24 | Neighbourhood Brief Observation Tool (NBOT) | Community/ Streetscape | Urban | Children/ Adolescents | North America, USA | **Neighborhood-level reliability** between 0.93 and 0.96  **Construct validity**  Correlations between physical incivilities scale and neighborhood poverty, crime density and residents’ perceptions of neighborhood conditions | (29) |
| 25 | Neighbourhood Active Living Potential (NALP) | Community/ Streetscape | Urban | Unspecific | North America, Canada | **Inter-rater reliability**  Mean of activity-friendliness items: 0.78  Mean of safety items: 0.76  Mean of density of destinations items: 0.83  **Internal consistency**  Activity-friendliness: 0.80 / 0.84  Safety: 0.77 / 0.82  Density of destinations: 0.87 / 0.91  **Convergent validity**  Safety of the environment was positively associated with neighborhood affluence, density of destinations was negatively associated with affluence and positively associated with higher proportions of persons in the neighborhood walking to work | (30, 31) |
| 26 | Neighbourhood Attribute Inventory (NAI) | Community/ Streetscape | Urban | Unspecific | North America, USA | **Inter-rater reliability**  83% agreement  **Internal consistency**  Physical incivility scale: Cronbach’s Alpha = 0.81  Social spaces scale: Cronbach’s Alpha = 0.61  Territoriality scale: Cronbach’s Alpha = 0.56  Correlations between scales and Census variables like poverty and low education | (32) |
| 27 | Neighbourhood Observational Checklist (NOC) | Community/ Streetscape | Urban | Unspecific | North America, USA | **Intra-rater reliability**  61% of items with substantial to almost perfect reliability (kappa 0.60-1.00); 29% moderate (0.40-0.59).  **Inter-rater reliability**  43% of items with substantial to almost perfect reliability (kappa 0.60-1.00); 25% moderate (0.40-0.59). | (33, 34) |
| 28 | Neighborhood Walkability Assessment (NWA) | Community/ Streetscape | Urban | Children/ Adolescents | North America, USA | None reported | (35) |
| 29 | Neighborhood Sidewalk Assessment Tool (NSAT) | Community/ Streetscape | Urban | Unspecific | Asia, Malaysia | **Internal consistency**  Cronbach’s Alpha ≥ 0.7 for all scales | (36) |
| 30 | Older People’s Environments and CVD Risk (OPECR) | Community/ Streetscape | Urban | Seniors | Europe, UK | **Inter-rater reliability**  ICCs between 0.58 and 0.99  Cohen’s Kappa between 0.56 and 0.96  **Criterion validity**  Good agreement between foot-based and virtual data collection | (37) |
| 31 | Older People’s External Residential Assessment Tool (OPERAT) | Community/ Streetscape | Urban | Seniors | Europe, Wales | **Construct validity**  Correlation between OPERAT items and corresponding questionnaire items; significant associations between 21 of 40 items  **Convergent validity**  Correlation between OPERAT domains, residents’ perceptions of the area and deprivation at postcode level  **Inter-rater reliability**  Krippendorff’s Alpha > 0.8 for all items | (38) |
| 32 | Pedestrian Environment Data Scan (PEDS) | Community/ Streetscape | Urban | Unspecific | North America, USA | **Inter-rater reliability**  Cohen’s kappa between 0.085 and 1.0  Observed agreement between 0.5 and 1.0.  **Intra-rater reliability**  Cohen’s kappa between 0.022 and 1.0  Percent agreement between 0.529 and 1.0 | (39) |
| 33 | Pedestrian Environment Quality Index (PEQI) | Community/ Streetscape | Urban | Unspecific | North America, USA | None reported | (40) |
| 34 | Pittsburgh Hill/Homewood Research on Neighborhood Change and Health (PHRESH Audit Tool) | Community/ Streetscape | Urban | Unspecific | North America, USA | **Inter-rater reliability**  Between 83.5% and 93.8% of items with excellent or good agreement | (41) |
| 35 | Physical Activity and Nutrition Features audit tool | Community/ Streetscape | Unspecific | Unspecific | North America, Canada | None reported | (42) |
| 36 | Pregnancy Infection and Nutrition (PIN3) Neighborhood Audit Instrument | Community/ Streetscape | Unspecific | Unspecific | North America, USA | **Intra-rater reliability**  Arterial or thoroughfare: Spearman correlation coefficient = 0.96 in urban and 0.96 in rural areas  Walkable neighborhood: Spearman correlation coefficient = 0.79 in urban and 0.82 in rural areas  Physical incivilities: Spearman correlation coefficient = 0.78 in urban and 0.77 in rural areas  Decoration: Spearman correlation coefficient = 0.50 in urban and 0.39 in rural areas  **Internal consistency**  Physical incivilities: Cronbach’s Alpha = 0.57 in urban and 0.59 in rural areas  Territoriality: Cronbach’s Alpha = 0.22 in urban and 0.34 in rural areas  Social spaces: Cronbach’s Alpha = 0.31 in urban and 0.24 in rural areas  Safety: Cronbach’s Alpha = 0.39 in urban and 0.41 in rural areas  Aesthetics/Streetscape: Cronbach’s Alpha = 0.29 in urban and 0.27 in rural areas  Functionality: Cronbach’s Alpha = 0.18 in urban and 0.11 in rural areas  Destinations: Cronbach’s Alpha = 0.27 in urban and 0.11 in rural areas | (43) |
| 37 | Rural Active Living Assessment (RALA) | Community/ Streetscape | Rural | Unspecific | North America, USA | Street Segment Assessment  **Inter-rater reliability**  Mean observed agreement: 0.919.  Mean Cohen’s kappa: 0.78 | (44) |
| 38 | Revised Residential Environment Assessment Tool (REAT) | Community/ Streetscape | Unspecific | Unspecific | Europe, Wales | **Inter-rater reliability**  Cohen’s kappa between 0.77 and 1.00  **Convergent validity**  Strong correlation between the street level neighborhood condition component and the PIN3 neighborhood condition scale  **Construct validity**  Associations with residents’ perceptions, e.g., residents living in neighborhoods with better street-level neighborhood condition perceived fewer incivilities and maintenance issues in their neighborhood.  **Predictive validity**  Associations between the overall REAT score and neighborhood attachment | (45) |
| 39 | Rural Pedestrian Environmental Audit Tool | Community/ Streetscape | Rural | Unspecific | North America, USA | **Inter-rater reliability**  > 80% agreement for each domain  Mean Cohen’s Kappa:  Destinations: 0.90  Street characteristics: 0.80  Quality of pedestrian facility: 0.45  Aesthetics/Quality of environment: 0.53  Social/dynamic environment: 0.50 | (46) |
| 40 | Sidewalk Assessment Tool | Community/ Streetscape | Urban | Unspecific | North America, USA | **Inter-rater reliability**  Cohen’s Kappa between 0.41 and 0.72 | (47) |
| 41 | Systematic Pedestrian and Cycling Environmental Scan (SPACES) / Madrid-SPACES / New Zealand-SPACES | Community/ Streetscape | Urban | Unspecific | Australia/ New Zealand/ Europe, Spain | SPACES  **Intra-rater reliability**  69 of 71 items with ≥ 70% agreement; 17 items with excellent (Kappa > 0.75), 47 with fair to good (0.4-0.75), and 7 with poor (< 0.4) reliability  **Inter-rater reliability**  45 of 67 items with ≥ 70% agreement; 21 items with excellent (Kappa > 0.75), 27 with fair to good (0.4-0.75), and 19 with poor (< 0.4) reliability  M-SPACES  **Intra-rater reliability**  ICCs between 0.00 and 0.92  **Inter-rater reliability**  ICCs between 0.02 and 0.79  NZ-SPACES  Acceptable level of agreement (ICC ≥ 0.70) between in-person and virtual audits | (48–50) |
| 42 | SPACES for Alleys | Community/ Streetscape | Urban | Unspecific | North America, USA | **Inter-rater reliability**  58 of 72 items with at least substantial agreement | (51) |
| 43 | Chicaco Community Adult Health Study (CCAHS) Systematic Social Observation Coding Sheet | Community/ Streetscape | Urban | Unspecific | North America, USA | Agreement between Systematic Social Observation and publicly available data from aerial photography: between 59% (Kappa = 0.19) and 90% (Kappa = 0.60) | (52, 53) |
| 44 | St. Louis Audit Tools (Analytic Audit Tool, Checklist Audit Tool) | Community/ Streetscape | Urban | Unspecific | North America, USA | **Inter-rater reliability**  Analytic: observed agreement between 0.42 and 0.92; ICC between 0.19 and 0.77  Checklist: observed agreement between 0.53 and 0.94; Cohen’s Kappa between 0.07 and 0.69 | (54) |
| 45 | Street Design Environmental Audit | Community/ Streetscape | Urban | Children/ Adolescents | North America, USA | None reported | (55) |
| 46 | SPOTLIGHT field audit tool and SPOTLIGHT virtual audit tool (S-VAT) | Community/ Streetscape | Urban | Unspecific | Europe | S-VAT  Intra-rater reliability  Between 91.7% (Kappa = 0.654) and 100% (Kappa = 1.0) agreement  **Inter-rater reliability**  Between substantial (78.6%; Kappa = 0.440) and high (99.2%; Kappa = 0.579) agreement  **Criterion validity**  Agreement between in-person and virtual audits between 87.3 and 99.9% | (56) |
| 47 | Stakeholders Walkability/Wheelability Audit in Neighbourhood for People with Disabilities (SWAN/SWAN-PWD) | Community/ Streetscape | Urban | People with disabilities | North America, Canada | None reported | (57) |
| 48 | Scottish Walkability Assessment Tool (SWAT) | Community/ Streetscape | Urban | Unspecific | Europe, Scotland | **Intra-rater reliability**  17 items with very good reliability (kappa ≥ 0.7), 9 with fair to good reliability (kappa = 0.4-0.7), 3 items with poor reliability (kappa < 0.4)  **Inter-rater reliability**  15 items with very good reliability (kappa ≥ 0.7), 18 with fair to good reliability (kappa = 0.4-0.7), 15 items with poor reliability (kappa < 0.4) | (58) |
| 49 | Senior Walking Environmental Assessment Tool (SWEAT) / SWEAT-R (modified) | Community/ Streetscape | Urban | Seniors | North America, USA | SWEAT-R  **Intra-rater reliability**  75% of items with good or excellent reliability (kappa > 0.60)  **Inter-rater reliability**  88% of items with good or excellent reliability (kappa > 0.60) | (59) |
| 50 | The Walking Suitability Index of the Territory (T-WSI) | Community/ Streetscape | Urban | Unspecific | Europe, Italy | **Inter-rater reliability**  Overall ICC = 0.89 | (60) |
| 51 | WABSA (Walking Suitability Assessment Form / Bicycle Suitability Assessment Form) | Community/ Streetscape | Urban | Unspecific | North America, USA | Walking Suitability Assessment Form  **Inter-rater reliability**  Overall score: ICC = 0.79  **Criterion-related validity** between r = 0.15 and r = 0.84  Bicycle Suitability Assessment Form  **Inter-rater reliability**  Overall score: ICC = 0.90  **Criterion-related validity**  Overall score: r = 0.62 | (61) |
| 52 | Wisconsin Assessment of the Social and Built Environment (WASABE) | Community/ Streetscape | Unspecific | Unspecific | North America, USA | **Inter-rater reliability**  Percent agreement between 54% and 100%; 70% of items with more than 90% agreement | (62) |
| 53 | WalkBoston Walk Audit Form | Community/ Streetscape | Urban | Unspecific | North America, USA | None reported | (63, 64) |
| 54 | Walking Environment Audit Tool-Diabetes (WEAT-D) | Community/ Streetscape | Unspecific | People with diabetes | North America, USA | None reported | (65) |
| 55 | Walking Route Audit Tool (WRATS) | Community/ Streetscape | Urban | Seniors | North America, USA | **Inter-rater reliability**  Kappa between 0.13 and 1.0; 27 of 44 items with Kappa ≥ 0.60 or ≥ 75% agreement | (66) |
| 56 | BRAT Direct Observation | Parks/Trails/ Public open spaces | Urban | Unspecific | North America, USA | **Inter-rater reliability**  Average agreement within domains between 83.7% and 91.9%  **Validity**  Agreement with “gold standard” assessment by consensus of two investigators involved in the tool development: average between 63.3% and 88.3% | (67) |
| 57 | Bridging the Gap Community Obesity Measures Project (BTG-COMP) - Park Observation Form | Parks/Trails/ Public open spaces | Urban | Children/ Adolescents | North America, USA | **Inter-rater reliability**  57% of items with almost perfect or substantial reliability, 35% with moderate or fair reliability, 8% with poor reliability | (68, 69) |
| 58 | Community Park Audit Tool (CPAT) | Parks/Trails/ Public open spaces | Urban | Unspecific | North America, USA | **Inter-rater reliability**  126 of 130 items with > 70% agreement  66 of 74 items with at least moderate reliability (kappa ≥ 0.4) | (70) |
| 59 | CLAN Public Open Space Audit Tool (C-POST) | Parks/Trails/ Public open spaces | Urban | Children/ Adolescents | Australia | All items showed at least adequate intra- and inter-rater reliability | (71) |
| 60 | Environmental Assessment of Public Recreation Spaces (EAPRS) / Abbreviated / Mini | Parks/Trails/ Public open spaces | Urban | Unspecific | North America, USA | **Inter-rater reliability**  69.6% of items with good-excellent range or high percent agreement | (72) |
| 61 | Natural Environment Scoring Tool (NEST) | Parks/Trails/ Public open spaces | Urban | Unspecific | Europe, England, Lithuania, Nether-lands, Spain | **Inter-rater reliability**  R-values between 0.76 and 0.96; percent agreement between 80% and 83% | (73) |
| 62 | Neighbourhod Green Space Tool (NGST) | Parks/Trails/ Public open spaces | Urban | Unspecific | Europe, England | **Inter-rater reliability**  ICC for overall score: 0.727  ICCs for domains between 0.575 and 0.948 | (74) |
| 63 | Parks and Play Spaces Audit | Parks/Trails/ Public open spaces | Urban | Children/ Adolescents | North America, USA | None reported | (75) |
| 64 | Path Environment Audit Tool (PEAT) | Parks/Trails/ Public open spaces | Unspecific | Unspecific | North America, USA | **Inter-rater reliability**  Percent agreement between 34% and 100%  ICCs between -0.04 and 0.84  Kappa between -0.03 and 1.0 | (76) |
| 65 | Public Open Space Tool (POST) / NZ-POST / New Remote POST | Parks/Trails/ Public open spaces | Urban | Unspecific | Australia/ New Zealand | POST  **Inter-rater reliability**  Kappa between 0.6 and 1.0  **Content validity** assessed by a panel of six experts.  **Agreement between remote and in-person assessment:**  17 items with substantial agreement (kappa > 0.6), 15 items with moderate agreement (0.6 > kappa > 0.4), 9 items with poor agreement  NZ-POST  Adequate reliability | (77–80) |
| 66 | READI Park Audit Tool | Parks/Trails/ Public open spaces | Unspecific | Unspecific | Australia | **Intra-rater reliability**  Percent agreement between 70% and 100%  **Inter-rater reliability**  Percent agreement between 82% and 100% | (81) |
| 67 | Systematic Audit of Green-Space Environments (SAGE) | Parks/Trails/ Public open spaces | Urban | Unspecific | North America, USA | None reported | (82) |
| 68 | Woods in and Around Towns (WIAT) | Parks/Trails/ Public open spaces | Urban | Unspecific | Europe, Scotland | None reported | (83) |
| 69 | Americas Playgrounds Safety Report Card | Playgrounds/ Recreation Facilities | Urban | Children/ Adolescents | North America, USA | **Inter-rater reliability**  was found to be acceptable (ICC = 0.77) | (84) |
| 70 | Bridging the Gap Community Obesity Measures Project (BTG-COMP) PA Facility Observation Form | Playgrounds/ Recreation Facilities | Urban | Children/ Adolescents | North America, USA | None reported | (85) |
| 71 | Physical Activity Resource Assessment Tool (PARA) | Playgrounds/ Recreation Facilities | Urban | Unspecific | North America, USA | Reliability tests of a 10% overlap showed good reliability (r s > .77) | (86) |
| 72 | The Parks, Activity and Recreation among Kids (PARK) Tool | Playgrounds/ Recreation Facilities | Urban | Children/ Adolescents | North America, Canada | **Intra-rater reliability**  Kappa > 0.4 for all but 4 items  **Inter-rater reliability**  86% of items with ≥ 75% agreement  85% of items with kappa > 0.4 (28% moderate, 27% substantial, 30% almost perfect) | (87) |
| 73 | Play Space Audit Tool (PSAT) | Playgrounds/ Recreation Facilities | Urban | Children/ Adolescents | North America, USA | **Inter-rater reliability**  Percent agreement between 67% and 100%  Kappa between 0.09 and 1.00 | (88) |
| 74 | Recreational Facility Audit Tool (RecFAT) | Playgrounds/ Recreation Facilities | Urban | Unspecific | Asia, Hong Kong | **Intra-rater reliability**  Mean percent agreement: 96.9% (range 91.0% - 100%)  76% of items with good kappa values, 4 items with moderate, 5 with poor  **Inter-rater reliability**  Mean percent agreement: 90.6% (range 80.0% - 100%)  51% of items with good kappa values, 12 items with moderate, 12 with poor  **Internal consistency**  Cronbach’s Alpha between 0.45 and 0.85 | (89) |
| 75 | Recreation Facility Evaluation Tool (RFET) | Playgrounds/ Recreation Facilities | Urban | Unspecific | North America, USA | **Inter-rater reliability**  Overall, the items were reliable | (90) |
| 76 | Computer Assisted Neighborhood Visual Assessment (CANVAS)/ “Drop-and-Spin Neighborhood Auditing” | Online-Tool/ Software | Urban | Unspecific | North America, USA | **Inter-rater reliability**  64 of 187 items with kappa or ICC scores above 0.80 and an additional 33 with scores above 0.60 | (91) |
| 77 | Complete Streets Assessment Tool (CSAT) | Online-Tool/ Software | Urban | Unspecific | North America, USA | None reported | (92) |
| 78 | Environmental Google Street View based Cycling (EGA-Cycling) | Online-Tool/ Software | Urban | Children/ Adolescents | Europe, Belgium | **Intra-rater reliability**  Cohen’s kappa between 0.47 and 1.00.  Observed agreement between 0.80 and 1.00.  **Inter-rater reliability**  Cohen’s kappa between -0.03 and 1.00.  Observed agreement between 0.367 and 1.00.  **Criterion validity**  Kappa values between virtually assessed and on-site assessed items between -0.06 and 1.00.  Observed agreement between 0.30 and 1.00. | (93) |
| 79 | ESRI Survey Audit Tool (ArcGIS) | Online-Tool/ Software | Unspecific | Seniors | Australia | None reported | (94) |
| 80 | Pedestrian Environment Review System (PERS) / Cycling Environment Review System (CERS) / Forty Area Study Street View (FASTVIEW) | Online-Tool/ Software | Urban | Unspecific | Europe, UK | **Intra-rater reliability**  Mean observed agreement: 0.81 (Kappa = 0.4)  **Inter-rater reliability**  Mean observed agreement: 0.717.  **Criterion reliability**  Mean observed agreement between in-person and desk-based audits: 0.84 (range 0.75-0.97; Kappa = 0.5-0.9) | (95) |
| 81 | Neighborhood Disorder Observational Scale | Online-Tool/ Software | Urban | Unspecific | Europe, Spain | **Inter-rater reliability**  Average of all items: fair agreement (0.21-0.40)  Mean of each subscale: moderate agreement (0.41-0.60)  **Criterion validity**  Correlation between physical and virtual audits | (96) |
| 82 | Public Open Space Desktop Auditing Tool (POSDAT) | Online-Tool/ Software | Urban | Unspecific | Australia | **Inter-rater reliability**  24 items with substantial reliability (kappa ≥ 0.6), 8 with moderate reliability (0.4 ≥ kappa < 0.6), and 11 with poor reliability (kappa < 0.4)  **Criterion validity**  39 items with substantial agreement (kappa ≥ 0.6) and one with moderate agreement (0.4 ≥ kappa < 0.6) between in-person and virtual audit | (97) |
| 83 | Systematic Social Observation Instrument (SSOI) | Online-Tool/ Software | Urban | Unspecific | North America, Canada | **Inter-rater reliability**  Average ICC across all items in 2011 was r = 0.85, in 2012, r = 0.72 and in 2013, r = 0.71  **Internal consistency**  2011: Cronbach’s alpha = 0.73; 2012: 0.64; 2013: 0.72 | (98) |
| 84 | Stanford Healthy Neighborhood Discovery Tool | Online-Tool/ Software | Urban | Seniors | North America, USA | **Inter-rater reliability** of coding schema: observed agreement > 90%; PABAK > 0.80.  Coding schema reviewed for construct validity | (99) |
| 85 | Virtual Audits of Streetscapes by Crowdworkers | Online-Tool/ Software | Urban | Unspecific | Asia, Japan | **Inter-rater reliability**  Kappa/ICC between 0.06 and 0.97; observed agreement between 0.73 and 0.988.  **Agreement between in-person and virtual audits**  Kappa/ICC between 0.30 and 0.99; observed agreement between 0.807 and 0.995. | (100) |
| 86 | Virtual-Systematic Tool for Evaluating Pedestrian Streetscapes (Virtual-STEPS) | Online-Tool/ Software | Urban | Unspecific | North America, Canada | **Inter-rater reliability**  75% of items with an observed agreement above 80%.  42.5% of items with almost perfect reliability (Kappa or ICC > 0.80), 27.5% of items with substantial reliability (Kappa or ICC 0.61-0.80), 15% of items with moderate reliability (Kappa or ICC 0.41-0.60)  **Criterion validity**  80% of items with an observed agreement above 80% between in-person and virtual audits.  50.0% of items with almost perfect agreement (Kappa or ICC > 0.80), 32.5% of items with substantial agreement (Kappa or ICC 0.61–0.80), 15.0% of items with moderate agreement (Kappa or ICC 0.41–0.60) | (101) |
| 87 | Space for children’s play inventory of the living environment [Raum für Kinderspiel Wohnumfeldinventar] | Community/ Streetscape | Urban | Children/ Adolescents | Europe, Germany | None reported | (102) |
| 88 | Integrated Microscale Walk Audit [Integriertes Walk-Audit auf Mikroebene (IWAM)] | Community/ Streetscape | Urban | Unspecific | Europe, Germany | None reported | (103) |
| 89 | Walkability Checklist [Checkliste/Prüfliste für Fußgängerfreundlichkeit] | Community/ Streetscape | Urban | Unspecific | Europe, Germany | None reported | (104, 105) |
| 90 | Playground Check [Spielplatz-Check] | Playgrounds/ Recreation Facilities | Unspecific | Children/ Adolescents | Europe, Germany | None reported | (106) |

Supplementary table 2: Summary of the results of systematic reviews on associations between the built environment and physical activity (children and adolescents)

| **Nordbo et al. 2020: Promoting activity participation and well-being among children and adolescents: a systematic review of neighborhood built-environment determinants (107) (age: 5-18 years)** | | | | | | | | |
| --- | --- | --- | --- | --- | --- | --- | --- | --- |
| **Unspecified PA** | **MVPA** | **Leisure-time PA** | **Leisure-time walking/ cycling** | **Active travel** | **Outdoor play/activity** | **Organized sports** | **Wellbeing** | **Use of public open spaces** |
| Road/street pattern and connectivity (+) |  | Facility and amenity index (+) |  | Less traffic and/or higher safety (+) | Less traffic and/or higher safety (+) |  | Increased count/ proportion of green/open space (+) |  |
| Facility and amenity index (+) |  |  |  | Increased traffic exposure and/or safety concerns (-) | Increased count/ proportion of facilities/ amenities (+) |  | Less favorable esthetics (-) |  |
|  |  |  |  | Pedestrian infrastructure (+) |  |  |  |  |
|  |  |  |  | Walkability (+) |  |  |  |  |
|  |  |  |  | Shorter Distance to facilities/ amenities (+) |  |  |  |  |
| **Lambert et al. 2019: What Is the Relationship between the Neighbourhood Built Environment and Time Spent in Outdoor Play? A Systematic Review (108) (age: 0-18 years)** | | | | | | | | |
|  |  |  |  |  | Lower traffic volumes (+) (6-11 years) |  |  |  |
|  |  |  |  |  | Yard access (+) (3-10 years) |  |  |  |
|  |  |  |  |  | Increased neighborhood greenness (+) (2-15 years) |  |  |  |
|  |  |  |  |  | Traffic-calming street features (+) (limited evidence) |  |  |  |
|  |  |  |  |  | Low traffic speeds (+) (limited evidence) |  |  |  |
|  |  |  |  |  | Neighborhood disorder (+) (limited evidence) |  |  |  |
|  |  |  |  |  | Residential density (+) (limited evidence) |  |  |  |
| **Timperio et al. 2015: Playability: Built and Social Environment Features That Promote Physical Activity Within Children (109) (age: 5-13 years)** | | | | | | | | |
| Seeing others exercise in the neighborhood (+) |  |  |  | Proximity to school (+) |  |  |  |  |
|  |  |  |  | Neighborhood walking and cycling infrastructure (e.g. presence and quality of sidewalks) (+) |  |  |  |  |
|  |  |  |  | Pedestrian safety/crossing infrastructure (+) |  |  |  |  |
| **Larouche 2015: Built Environment Features that Promote Cycling in School-Aged Children (110) (age: 5-17 years)** | | | | | | | | |
|  |  |  |  | Increased distance between home and school (-) |  |  |  |  |
| **D'Haese et al. 2015: Cross-continental comparison of the association between the physical environment and active transportation in children: a systematic review (111) (age: 6-12 years)** | | | | | | | | |
|  |  |  |  | Walkability (+) (walking or cycling to school) |  |  |  |  |
|  |  |  |  | Density (+) (walking to school) |  |  |  |  |
|  |  |  |  | Accessibility (+) (walking to school) |  |  |  |  |
|  |  |  |  | Traffic safety (+) (possible association with all forms of active transportation to school) |  |  |  |  |
| **McGrath et al. 2015: Associations of Objectively Measured Built-Environment Attributes with Youth Moderate–Vigorous Physical Activity: A Systematic Review and Meta-Analysis (112) (age: 5-17 years)** | | | | | | | | |
|  | Play facilities, parks, playgrounds, and features that facilitate walking (+) (15 years) |  |  |  |  |  |  |  |
|  | Play facilities, parks, playgrounds, and features that facilitate walking (-) (9 years) |  |  |  |  |  |  |  |
| **Ding et al. 2011: Neighborhood Environment and Physical Activity Among Youth: A Review (113) (age: 3-18 years)** | | | | | | | | |
| Access to recreation facilities (children 5-12) |  |  |  |  |  |  |  |  |
| Land-use mix (children and adolescents) |  |  |  |  |  |  |  |  |
| Residential density (children + adolescents) |  |  |  |  |  |  |  |  |
| Walkability (children 5-12) |  |  |  |  |  |  |  |  |
| Walking/ biking facilities (children 5-12) |  |  |  |  |  |  |  |  |
| Traffic speed/volume (children 5-12) |  |  |  |  |  |  |  |  |
| Pedestrian safety structures (children 5-12) |  |  |  |  |  |  |  |  |
| Incivilities/ disorders (children 5-12) |  |  |  |  |  |  |  |  |
| Vegetation (children 5-12) |  |  |  |  |  |  |  |  |
| **Van Hecke et al. 2018: Public open space characteristics influencing adolescents’ use and physical activity: A systematic literature review of qualitative and quantitative studies (114) (age: 12-16 years)** | | | | | | | | |
|  |  |  |  |  |  |  |  | Lack of age-appropriate features (e.g., small swings and slides) (-) |
|  |  |  |  |  |  |  |  | sport- and adventurous playgrounds (+) |
|  |  |  |  |  |  |  |  | Presence of trails/walking paths (+) |
| **Ikeda et al. 2018: Associations of children's active school travel with perceptions of the physical environment and characteristics of the social environment: A systematic review (115) (age: 5-13 years)** | | | | | | | | |
|  |  |  |  | Safety (+) |  |  |  |  |
|  |  |  |  | Walkability (+) |  |  |  |  |
|  |  |  |  | Neighborhood social interactions (+) |  |  |  |  |
|  |  |  |  | Travel distance (-) |  |  |  |  |
|  |  |  |  | Car ownership (-) |  |  |  |  |
| **Marzi et al. 2018: Social and physical environmental correlates of independent mobility in children: a systematic review taking sex/gender differences into account (116) (age: 3-12 years)** | | | | | | | | |
|  |  |  |  | Neighborhood safety (+) |  |  |  |  |
|  |  |  |  | Perception of traffic |  |  |  |  |
|  |  |  |  | Car ownership (-) |  |  |  |  |
|  |  |  |  | Distance |  |  |  |  |
|  |  |  |  | Neighborhood design |  |  |  |  |

Supplementary table 3: Summary of the results of systematic reviews on associations between the built environment and physical activity (adults)

| **McCormack & Shiell 2011: In search of causality: a systematic review of the relationship between the built environment and physical activity among adults (117)** | | | | | | |
| --- | --- | --- | --- | --- | --- | --- |
| **Unspecified PA** | **Leisure-time PA** | **Total walking/cycling** | **Leisure-time walking/cycling** | **Active travel** | **Walking for transportation** | **Cycling for transportation** |
| Land-use mix (+) |  |  |  |  |  |  |
| Connectivity (+) |  |  |  |  |  |  |
| Population density (+) |  |  |  |  |  |  |
| Overall neighborhood design (+) |  |  |  |  |  |  |
| **Van Holle et al. 2012: Relationship between the physical environment and different domains of physical activity in European adults: a systematic review (118) (age: 18-65 years)** | | | | | | |
| Walkability (+) | Quality of the environment (+) (possible evidence) | Urbanization degree (+) (possible evidence) | Traffic-related safety (+) | Access to shops/services/ work (+) | Walkability (+) | Walkability (+) |
| Quality of the environment (+) |  |  |  | Walking/cycling facilities (+) (possible evidence) |  | Access to shops/services/ work (+) |
| Urbanization degree (-) |  |  |  |  |  | Urbanization degree (+) |
| Access to recreation facilities (+) (possible evidence) |  |  |  |  |  | Walking/cycling facilities (+) (possible evidence) |
|  |  |  |  |  |  | Hilliness (-) (possible evidence) |
| **Wang & Wen 2017: The Relationship between the Neighborhood Built Environment and Active Transportation among Adults: A Systematic Literature Review (119)** | | | | | | |
|  |  |  |  |  | Residential density (+) | Street connectivity (+) |
|  |  |  |  |  | Land use mix (+) | Bike lane (+) |
|  |  |  |  |  | Street connectivity (+) | Neighborhood aesthetics (-) |
|  |  |  |  |  | Retail land use (+) | Access to destinations (-) |
|  |  |  |  |  | Walkability (+) |  |
|  |  |  |  |  | Sidewalk (+) |  |
|  |  |  |  |  | Assess to destinations (+) |  |
| **Carlin et al. 2017: A life course examination of the physical environmental determinants of physical activity behaviour: A "Determinants of Diet and Physical Activity" (DEDIPAC) umbrella systematic literature review (120)** | | | | | | |
| Negative street characteristics (-) |  | Street connectivity (+) |  | Street connectivity (+) |  |  |
| Walkability (+) (limited evidence) |  | Land use diversity (+) |  | Land use diversity (+) |  |  |
| Level of urbanization (+) |  | Availability/access/ proximity of public transport system (+) |  |  |  |  |

Supplementary table 4: Summary of the results of systematic reviews on associations between the built environment and physical activity (older adults)

| **Bonaccorsi et al. 2020: Impact of the Built Environment and the Neighborhood in Promoting the Physical Activity and the Healthy Aging in Older People: An Umbrella Review (121) (age: > 65 years)** | | | |
| --- | --- | --- | --- |
| **Unspecified PA** | **Leisure-time PA** | **Walking** | **Active travel** |
| Walkability (+) | Walkability (+) | Walkability (+) | Walkability (+) |
| Land-use mix (+) | Land-use mix - access (+) | Residential density/urbanization - density of physical activity facilities (+) | Residential density/urbanization (+) |
| Street connectivity (+) | Aesthetically pleasing scenery (+) | Street connectivity (+) | Overall access to facilities, destinations, and services (+) |
| Overall access to facilities (+) | Access to public transit (+) | Access to shops/commercial (+) | Land use-mix - destination diversity (+) |
| Access to shops/commercial (+) | Access to recreational facilities (+) | Access to public transport (+) | Access to shops/commercial (+) |
| Poor pedestrian access to shopping centers (-) | Access to park/open space (+) | Access to nature/parks/open space (+) | Access to food outlets (+) |
| Access to public transport (+) | Barriers to walking/cycling (-) | Pedestrian-friendly infrastructure (+) | Access to business/ institutional/industrial destinations (+) |
| Access to nature/parks/open space (+) |  | Greenery/aesthetically pleasing scenery (+) | Access to public transport (+) |
| Access to recreational facilities (+) |  | Crime-related safety (+) | Access to parks/open space/ recreation (+) |
| Acces to places for social interaction (+) |  |  | Pedestrian-friendly infrastructure - footpath quality, pedestrian crossing (+) |
| Access to exercise opportunities (senior oriented group activities) (+) |  |  | Pedestrian/cycling facilities (+) |
| Access to rest areas - seating, benches, public washrooms |  |  | Availability of benches/sitting facilities (+) |
| Pedestrian-friendly infrastructure - footpath quality, lack of hills, sidewalk characteristics (presence and continuity, quality and maintenance, slopes and curbs, temporary obstacles on sidewalks), separation between pedestrians and other nonmotorized transport (+) |  |  | Street lighting (+) |
| Poor pedestrian-friendly infrastructure/footpath quality (-) |  |  | Easy access to building entrance, whelchair access, walking access (+) |
| Pedestrian/cycling facilities (+) |  |  | Human and motorized traffic volume (+) |
| Aesthetics - Greenery/Aesthetically pleasing scenery, buildings, and streetscape/natural scenery (+) |  |  | Littering/vandalism/decay (-) |
| Lack of aesthetically pleasing scenery (-) |  |  |  |
| Crime-related safety - street lighting (+) |  |  |  |
| Crime-related safety - unattended dogs, inadequate lighting, lack of street lighting and upkeep (-) |  |  |  |
| Traffic-related safety - zebra-crossing characteristics, signaled crosswalks (+) |  |  |  |
| Traffic (-) |  |  |  |
| High environmental quality (+) |  |  |  |
| Pollution (-) |  |  |  |
| Noise (-) |  |  |  |

Supplementary table 5: Summary of the results of systematic reviews on associations between the built environment and physical activity (no specific age group)

| **Schulz et al. 2016: Built environment and health: a systematic review of studies in Germany (122)** | | | | | | |
| --- | --- | --- | --- | --- | --- | --- |
| **Unspecified PA** | **MVPA** | **Leisure-time PA** | **Walking** | **Cycling** | **Active travel** | **Cycling for transportation** |
| Better street connectivity/walkability (+) | Close distance to activity-related destinations (playgrounds and sports facilities) (+) |  | Better street connectivity/walkability (+) |  | Close distance to sports-unrelated destinations (i.e. transit) (+) |  |
| **Yang et al. 2019: Towards a cycling-friendly city: An updated review of the associations between built environment and cycling behaviors (2007–2017) (123)** | | | | | | |
|  |  |  |  | Presence of cycling routes/paths (+) |  | Street connectivity (+) |
|  |  |  |  | Open space and green space (+) |  | Availability of non-residential destinations (+) |
|  |  |  |  | Aesthetics and attractiveness (+) |  | Presence of cycling routes/paths (+) |
|  |  |  |  | Terrain slope (emerging) |  | Land use mix (+) (emerging) |
|  |  |  |  | Cycling safety design features (emerging) |  | Availability of green spaces (+) (emerging) |
|  |  |  |  |  |  |  |
| **Kärmeniemi et al. 2018: The Built Environment as a Determinant of Physical Activity: A Systematic Review of Longitudinal Studies and Natural Experiments (124)** | | | | | | |
| Accessibility (+) |  |  |  |  | Accessibility (+) |  |
| Infrastructure for walking/cycling (+) |  |  |  |  | Infrastructure for walking/cycling (+) |  |
| Public transport (+) |  |  |  |  | Public transport (+) |  |
| Aesthetics (+) |  |  |  |  |  |  |
| Safety (+) |  |  |  |  |  |  |

Literature Cited

1. Hoehner CM, Ivy A, Ramirez LKB, Handy S, Brownson RC. Active neighborhood checklist: a user-friendly and reliable tool for assessing activity friendliness. Am J Health Promot 2007; 21(6):534–7.

2. Andresen EM, Malmstrom TK, Wolinsky FD, Schootman M, Miller JP, Miller DK. Rating neighborhoods for older adult health: results from the African American Health study. BMC Public Health 2008; 8(1):35.

3. Curl A, Thompson CW, Aspinall P, Ormerod M. Developing an audit checklist to assess outdoor falls risk. Proc Inst Civ Eng Urban Des Plan 2016; 169(3):138–53.

4. Hoedl S, Titze S, Oja P. The bikeability and walkability evaluation table reliability and application. Am J Prev Med 2010; 39(5):457–9.

5. Slater SJ, Nicholson L, Chriqui J, Barker DC, Chaloupka FJ, Johnston LD. Walkable communities and adolescent weight. Am J Prev Med 2013; 44(2):164–8.

6. Kealey M, Kruger J, Hunter R, Ivey S, Satariano W, Bayles C et al. Engaging older adults to be more active where they live: audit tool development. In: Proceedings of the 19th national conference on chronic disease prevention and control.

7. Perez Adriana, Garces Anamarie, Hunter Rebecca H., Marquez David X. An Audit of a Diverse Community for Safe Routes to Age in Place: Environmental Policy Implications. Journal of Gerontological Nursing 2015; 41(3):13–21.

8. Kroeger GL, Messer L, Edwards SE, Miranda ML. A novel tool for assessing and summarizing the built environment. Int J Health Geogr 2012; 11(1):46.

9. Aceves-González C, Ekambaram K, Rey-Galindo J, Rizo-Corona L. The role of perceived pedestrian safety on designing safer built environments. Traffic Injury Prevention 2020; 21(sup1):S84-S89.

10. Wong F, Stevens D, O'Connor-Duffany K, Siegel K, Gao Y. Community Health Environment Scan Survey (CHESS): a novel tool that captures the impact of the built environment on lifestyle factors. Glob Health Action 2011; 4:5276.

11. Su M, Du Y, Liu Q, Ren Y, Kawachi I, Lv J et al. Objective assessment of urban built environment related to physical activity — development, reliability and validity of the China Urban Built Environment Scan Tool (CUBEST). BMC Public Health 2014; 14(1):109.

12. Abley S, Wade-Brown C, Thomas L, Linton L, Shuttleworth K. Guide to undertaking community street reviews. Auckland, NZ Trabsport Agency 2010.

13. Kleopa D, Panayiotou A, Kouta C, Middleton N. Content validity of neighbourhood environment audit tool: virtual ethnographic study. Eur J Public Health 2018; 28(Suppl 4).

14. Cerin E, Chan K, Macfarlane DJ, Lee K, Lai P. Objective assessment of walking environments in ultra-dense cities: Development and reliability of the Environment in Asia Scan Tool—Hong Kong version (EAST-HK). Health Place 2011; 17(4):937–45. Available from: URL: https://www.sciencedirect.com/science/article/pii/S1353829211000645.

15. Chow CK, Corsi DJ, Lock K, Madhavan M, Mackie P, Li W et al. A Novel Method to Evaluate the Community Built Environment Using Photographs – Environmental Profile of a Community Health (EPOCH) Photo Neighbourhood Evaluation Tool. PLoS One 2014; 9(11):e110042.

16. Seguin RA, Lo BK, Sriram U, Connor LM, Totta A. Development and testing of a community audit tool to assess rural built environments: Inventories for Community Health Assessment in Rural Towns. Prev Med Rep 2017; 7:169–75.

17. Ghalichi L, Mohammad K, Majdzadeh R, Hoseini M, Pournik O, Nedjat S. Developing a reliable and valid instrument to assess health-affecting aspects of neighborhoods in Tehran. J Res Med Sci 2012; 17(6):552–6.

18. Nesoff ED, Milam AJ, Pollack KM, Curriero FC, Bowie JV, Gielen AC et al. Novel Methods for Environmental Assessment of Pedestrian Injury: Creation and Validation of the Inventory for Pedestrian Safety Infrastructure. J Urban Health 2018; 95(2):208–21.

19. Boarnet MG, Day K, Alfonzo M, Forsyth A, Oakes M. The Irvine–Minnesota Inventory to Measure Built Environments: Reliability Tests. American Journal of Preventive Medicine 2006; 30(2):153-159.e43. Available from: URL: https://www.sciencedirect.com/science/article/pii/S0749379705004290.

20. Millstein RA, Cain KL, Sallis JF, Conway TL, Geremia C, Frank LD et al. Development, scoring, and reliability of the Microscale Audit of Pedestrian Streetscapes (MAPS). BMC Public Health 2013; 13:403.

21. Cain KL, Gavand KA, Conway TL, Geremia CM, Millstein RA, Frank LD et al. Developing and Validating an Abbreviated Version of the Microscale Audit for Pedestrian Streetscapes (MAPS-Abbreviated). J Transp Health 2017; 5:84–96.

22. Sallis JF, Cain KL, Conway TL, Gavand KA, Millstein RA, Geremia CM et al. Is Your Neighborhood Designed to Support Physical Activity? A Brief Streetscape Audit Tool. Prev Chronic Dis 2015; 12:E141.

23. Cain KL, Geremia CM, Conway TL, Frank LD, Chapman JE, Fox EH et al. Development and reliability of a streetscape observation instrument for international use: MAPS-global. Int J Behav Nutr Phys Act 2018; 15(1):19.

24. Pocock T, Moore A, Molina-García J, Queralt A, Mandic S. School Neighbourhood Built Environment Assessment for Adolescents’ Active Transport to School: Modification of an Environmental Audit Tool and Protocol (MAPS Global-SN). International Journal of Environmental Research and Public Health 2020; 17(7).

25. Gamache S, Vincent C, Routhier F, McFadyen BJ, Fiset D. Measure of accessibility to urban infrastructures for adults with physical disabilities (MAUAP): inter-rater reliability study. Med Res Arch 2016; 4(5).

26. Suminski RR, Heinrich KM, Poston WSC, Hyder M, Pyle S. Characteristics of Urban Sidewalks/Streets and Objectively Measured Physical Activity. J Urban Health 2008; 85(2):178–90.

27. Ewing R, Handy S, Brownson RC, Clemente O, Winston E. Identifying and Measuring Urban Design Qualities Related to Walkability. Journal of Physical Activity and Health 2006; 3(s1):S223-S240. Available from: URL: https://journals.humankinetics.com/view/journals/jpah/3/s1/article-pS223.xml.

28. Burton EJ, Mitchell L, Stride CB. Good places for ageing in place: development of objective built environment measures for investigating links with older people's wellbeing. BMC Public Health 2011; 11(1):839.

29. Caughy MO, O’Campo PJ, Patterson J. A brief observational measure for urban neighborhoods. Health Place 2001; 7(3):225–36. Available from: URL: https://www.sciencedirect.com/science/article/pii/S1353829201000120.

30. Gauvin L, Richard L, Craig CL, Spivock M, Riva M, Forster M et al. From walkability to active living potential: an "ecometric" validation study. Am J Prev Med 2005; 28(2 Suppl 2):126–33.

31. Fuller DL, Muhajarine N. Replication of the Neighborhood Active Living Potential Measure in Saskatoon, Canada. American Journal of Preventive Medicine 2010; 39(4):364–7. Available from: URL: https://www.sciencedirect.com/science/article/pii/S0749379710003880.

32. Laraia BA, Messer L, Kaufman JS, Dole N, Caughy M, O'Campo P et al. Direct observation of neighborhood attributes in an urban area of the US south: characterizing the social context of pregnancy. Int J Health Geogr 2006; 5(1):11.

33. Schulz Amy J., Kannan Srimathi, Dvonch J. Timothy, Israel Barbara A., Allen Alex, James Sherman A. et al. Social and Physical Environments and Disparities in Risk for Cardiovascular Disease: The Healthy Environments Partnership Conceptual Model. Environmental Health Perspectives 2005; 113(12):1817–25.

34. Zenk SN, Schulz AJ, Mentz G, House JS, Gravlee CC, Miranda PY et al. Inter-rater and test–retest reliability: Methods and results for the neighborhood observational checklist. Health Place 2007; 13(2):452–65. Available from: URL: https://www.sciencedirect.com/science/article/pii/S1353829206000311.

35. Topmiller M, Jacquez F, Vissman AT, Raleigh K, Miller-Francis J. Partnering with youth to map their neighborhood environments: a multilayered GIS approach. Fam Community Health 2015; 38(1):66–76.

36. Aghaabbasi M, Moeinaddini M, Zaly Shah M, Asadi-Shekari Z. A new assessment model to evaluate the microscale sidewalk design factors at the neighbourhood level. Journal of Transport & Health 2017; 5:97–112. Available from: URL: https://www.sciencedirect.com/science/article/pii/S2214140516303061.

37. Pliakas T, Hawkesworth S, Silverwood RJ, Nanchahal K, Grundy C, Armstrong B et al. Optimising measurement of health-related characteristics of the built environment: Comparing data collected by foot-based street audits, virtual street audits and routine secondary data sources. Health Place 2017; 43:75–84. Available from: URL: https://www.sciencedirect.com/science/article/pii/S1353829216304361.

38. Burholt V, Roberts MS, Musselwhite CBA. Older People’s External Residential Assessment Tool (OPERAT): a complementary participatory and metric approach to the development of an observational environmental measure. BMC Public Health 2016; 16(1):1022.

39. Clifton KJ, Livi Smith AD, Rodriguez D. The development and testing of an audit for the pedestrian environment. Landsc Urban Plan 2007; 80(1):95–110.

40. San Francisco Department of Public Health. Pedestrian Environmental Quality Index: Street Auditor’s Training Manual; 2012.

41. Ghosh-Dastidar M, Hunter GP, Sloan JC, Collins RL, Richardson AS, Troxel W et al. An audit tool for longitudinal assessment of the health-related characteristics of urban neighborhoods: implementation methods and reliability results. BMC Public Health 2020; 20(1):1519.

42. Gasevic D, Vukmirovich I, Yusuf S, Teo K, Chow C, Dagenais G et al. A Direct Assessment of “Obesogenic”Built Environments: Challenges and Recommendations. Journal of Environmental and Public Health 2011; 2011:161574.

43. Evenson KR, Sotres-Alvarez D, Herring AH, Messer L, Laraia BA, Rodríguez DA. Assessing urban and rural neighborhood characteristics using audit and GIS data: derivation and reliability of constructs. Int J Behav Nutr Phys Act 2009; 6:44.

44. Yousefian A, Hennessy E, Umstattd MR, Economos CD, Hallam JS, Hyatt RR et al. Development of the Rural Active Living Assessment Tools: measuring rural environments. Prev Med 2010; 50(Suppl 1):S86-92.

45. Poortinga W, Calve T, Jones N, Lannon S, Rees T, Rodgers SE et al. Neighborhood quality and attachment: Validation of the revised residential environment assessment tool. Environment and behavior 2017; 49(3):255–82.

46. Scanlin K, Haardoerfer R, Kegler MC, Glanz K. Development of a Pedestrian Audit Tool to Assess Rural Neighborhood Walkability. Journal of Physical Activity and Health 2014; 11(6):1085–96. Available from: URL: https://journals.humankinetics.com/view/journals/jpah/11/6/article-p1085.xml.

47. Williams JE, Evans M, Kirtland KA, Cavnar MM, Sharpe PA, Neet MJ et al. Development and Use of a Tool for Assessing Sidewalk Maintenance as an Environmental Support of Physical Activity. Health Promotion Practice 2005; 6(1):81–8.

48. Pikora TJ, Bull FC, Jamrozik K, Knuiman M, Giles-Corti B, Donovan RJ. Developing a reliable audit instrument to measure the physical environment for physical activity. Am J Prev Med 2002; 23(3):187–94.

49. Gullón P, Badland HM, Alfayate S, Bilal U, Escobar F, Cebrecos A et al. Assessing Walking and Cycling Environments in the Streets of Madrid: Comparing On-Field and Virtual Audits. J Urban Health 2015; 92(5):923–39.

50. Badland HM, Opit S, Witten K, Kearns RA, Mavoa S. Can Virtual Streetscape Audits Reliably Replace Physical Streetscape Audits? J Urban Health 2010; 87(6):1007–16.

51. Seymour M, Reynolds KD, Wolch J. Reliability of an Audit Tool for Systematic Assessment of Urban Alleyways. Journal of Physical Activity and Health 2010; 7(2):214–23. Available from: URL: https://journals.humankinetics.com/view/journals/jpah/7/2/article-p214.xml.

52. Mair C, Diez Roux AV, Morenoff JD. Neighborhood stressors and social support as predictors of depressive symptoms in the Chicago Community Adult Health Study. Health Place 2010; 16(5):811–9.

53. King KE. A comparison of two methods for measuring land use in public health research: systematic social observation versus remote sensing–based coded aerial photography. SAGE Open 2015; 5(2):2158244015589438.

54. Brownson RC, Hoehner CM, Brennan LK, Cook RA, Elliott MB, McMullen KM. Reliability of 2 Instruments for Auditing the Environment for Physical Activity. Journal of Physical Activity and Health 2004; 1(3):191–208. Available from: URL: https://journals.humankinetics.com/view/journals/jpah/1/3/article-p191.xml.

55. Raja S, Booth J, Norton JT, Crowell B, Gouck J, Bonaro K. Promoting Active Commuting to School Through Environmental and Policy Supports in Buffalo, New York. Journal of Public Health Management and Practice 2015; 21. Available from: URL: https://journals.lww.com/jphmp/Fulltext/2015/05001/Promoting_Active_Commuting_to_School_Through.19.aspx.

56. Bethlehem JR, Mackenbach JD, Ben-Rebah M, Compernolle S, Glonti K, Bárdos H et al. The SPOTLIGHT virtual audit tool: a valid and reliable tool to assess obesogenic characteristics of the built environment. Int J Health Geogr 2014; 13:52.

57. Mahmood A, O’Dea E, Bigonnesse C, Labbe D, Mahal T, Qureshi M et al. Stakeholders Walkability/Wheelability Audit in Neighbourhoods (SWAN): User-led audit and photographic documentation in Canada. Disability & Society 2020; 35(6):902–25.

58. Millington C, Ward Thompson C, Rowe D, Aspinall P, Fitzsimons C, Nelson N et al. Development of the Scottish Walkability Assessment Tool (SWAT). Health Place 2009; 15(2):474–81.

59. Michael YL, Keast EM, Chaudhury H, Day K, Mahmood A, Sarte AF. Revising the senior walking environmental assessment tool. Prev Med 2009; 48(3):247–9.

60. D’Alessandro D, Valeri D, Appolloni L. Reliability of T-WSI to Evaluate Neighborhoods Walkability and Its Changes over Time. International Journal of Environmental Research and Public Health 2020; 17(21).

61. Emery J, Crump C, Bors P. Reliability and validity of two instruments designed to assess the walking and bicycling suitability of sidewalks and roads. Am J Health Promot 2003; 18(1):38–46.

62. Malecki KC, Engelman CD, Peppard PE, Nieto FJ, Grabow ML, Bernardinello M et al. The Wisconsin Assessment of the Social and Built Environment (WASABE): a multi-dimensional objective audit instrument for examining neighborhood effects on health. BMC Public Health 2014; 14(1):1–15.

63. WalkBoston. Walk Audit Form [cited 2023 Apr 6]. Available from: URL: https://walkboston.org/sites/default/files/WalkBoston%20Walk%20Audit%20Form%20v3.pdf.

64. Suminski RR, Jackson-Short C, Duckworth N, Plautz E, Speakman K, Landgraf R et al. Dover Micro Open Street Events: Evaluation Results and Implications for Community-Based Physical Activity Programming. Front Public Health 2019; 7:356.

65. Hosler AS, Gallant MP, Riley-Jacome M, Rajulu DT. Relationship between objectively measured walkability and exercise walking among adults with diabetes. Journal of Environmental and Public Health 2014; 2014.

66. Kerr J, Carlson JA, Rosenberg DE, Withers A. Identifying and promoting safe walking routes in older adults. Health 2012; 04(09):720–4.

67. Bedimo-Rung AL, Gustat J, Tompkins BJ, Rice J, Thomson J. Development of a Direct Observation Instrument to Measure Environmental Characteristics of Parks for Physical Activity. J Phys Act Health 2006; 3(s1):S176-S189.

68. Nelson DS, Nezich T, Antonakos CL, Dubowitz T, Clarke P, Colabianchi N. Reliability and validity of environmental audits using GigaPan® technology in parks. Prev Med Rep 2019; 13:293–7. Available from: URL: https://www.sciencedirect.com/science/article/pii/S2211335519300063.

69. Zenk SN, Slater SJ, Rashid S. Collecting Contextual Health Survey Data Using Systematic Observation. In: Johnson TP, editor. Handbook of health survey methods. Hoboken, New Jersey: John Wiley & Sons, Inc; 2015. p. 421–45 (Wiley handbooks in survey methodology).

70. Kaczynski AT, Stanis SAW, Besenyi GM. Development and testing of a community stakeholder park audit tool. American Journal of Preventive Medicine 2012; 42(3):242–9.

71. Crawford D, Timperio A, Giles-Corti B, Ball K, Hume C, Roberts R et al. Do features of public open spaces vary according to neighbourhood socio-economic status? Health Place 2008; 14(4):889–93.

72. Saelens BE, Frank LD, Auffrey C, Whitaker RC, Burdette HL, Colabianchi N. Measuring Physical Environments of Parks and Playgrounds: EAPRS Instrument Development and Inter-Rater Reliability. J Phys Act Health 2006; 3(s1):S190-S207.

73. Gidlow C, van Kempen E, Smith G, Triguero-Mas M, Kruize H, Gražulevičienė R et al. Development of the natural environment scoring tool (NEST). Urban Forestry & Urban Greening 2018; 29:322–33.

74. Gidlow CJ, Ellis NJ, Bostock S. Development of the neighbourhood green space tool (NGST). Landscape and Urban Planning 2012; 106(4):347–58.

75. Patton-López MM, Muñoz R, Polanco K, Olson B, Brown G, DeGhetto S. Redesigning a Neighborhood Park to Increase Physical Activity. Journal of Public Health Management and Practice 2015; 21:S101-S105.

76. Troped PJ, Cromley EK, Fragala MS, Melly SJ, Hasbrouck HH, Gortmaker SL et al. Development and reliability and validity testing of an audit tool for trail/path characteristics: the Path Environment Audit Tool (PEAT). J Phys Act Health 2006; 3(s1):S158-S175.

77. Giles-Corti B, Broomhall MH, Knuiman M, Collins C, Douglas K, Ng K et al. Increasing walking: how important is distance to, attractiveness, and size of public open space? American Journal of Preventive Medicine 2005; 28(2):169–76.

78. Taylor BT, Fernando P, Bauman AE, Williamson A, Craig JC, Redman S. Measuring the Quality of Public Open Space Using Google Earth. American Journal of Preventive Medicine 2011; 40(2):105–12.

79. Oliver M, Witten K, Kearns RA, Mavoa S, Badland HM, Carroll P et al. Kids in the city study: research design and methodology. BMC Public Health 2011; 11(1):587.

80. Badland HM, Keam R, Witten K, Kearns RA. Examining Public Open Spaces by Neighborhood-Level Walkability and Deprivation. Journal of Physical Activity and Health 2010; 7(6):818–24. Available from: URL: https://journals.humankinetics.com/view/journals/jpah/7/6/article-p818.xml.

81. Veitch J, Salmon J, Ball K, Crawford D, Timperio A. Do features of public open spaces vary between urban and rural areas? Prev Med 2013; 56(2):107–11.

82. Byrne J, Wolch J, Swift J, Ryne C. SAGE (Systematic Audit Of Green-Space Environments) 2005.

83. Ward Thompson C, Roe J. Protocol to be followed when using the WIAT Questionnaire and Environmental Audit Tools 2010.

84. Suminski R, Presley T, Wasserman JA, Mayfield CA, McClain E, Johnson M. Playground Safety is Associated With Playground, Park, and Neighborhood Characteristics. Journal of Physical Activity and Health 2015; 12(3):402–8. Available from: URL: https://journals.humankinetics.com/view/journals/jpah/12/3/article-p402.xml.

85. Chaloupka FJ, Johnston LD. Bridging the Gap: research informing practice and policy for healthy youth behavior. American Journal of Preventive Medicine 2007; 33(4):S147-S161.

86. Lee RE, Booth KM, Reese-Smith JY, Regan G, Howard HH. The Physical Activity Resource Assessment (PARA) instrument: Evaluating features, amenities and incivilities of physical activity resources in urban neighborhoods. International Journal of Behavioral Nutrition and Physical Activity 2005; 2(1):13.

87. Bird ME, Datta GD, van Hulst A, Kestens Y, Barnett TA. A reliability assessment of a direct-observation park evaluation tool: the Parks, activity and recreation among kids (PARK) tool. BMC Public Health 2015; 15:1–11.

88. Gustat J, Anderson CE, Slater SJ. Development and testing of a brief play space audit tool. J Phys Act Health 2020; 17(1):13–20.

89. Lee KY, Macfarlane DJ, Cerin E. Objective evaluation of recreational facilities: Development and reliability of the recreational facility audit tool. Journal of Park and Recreation Administration (Online Edition) 2013.

90. Cavnar MM, Kirtland KA, Evans MH, Wilson DK, Williams JE, Mixon GM et al. Evaluating the Quality of Recreation Facilities: Development of an Assessment Tool. Journal of Park & Recreation Administration 2004; 22(1).

91. Bader MDM, Mooney SJ, Lee YJ, Sheehan D, Neckerman KM, Rundle AG et al. Development and deployment of the Computer Assisted Neighborhood Visual Assessment System (CANVAS) to measure health-related neighborhood conditions. Health Place 2015; 31:163–72.

92. Schlossberg M, Brehm C. Participatory Geographic Information Systems and Active Transportation: Collecting Data and Creating Change. Transportation Research Record 2009; 2105(1):83–91.

93. Vanwolleghem G, van Dyck D, Ducheyne F, Bourdeaudhuij I de, Cardon G. Assessing the environmental characteristics of cycling routes to school: a study on the reliability and validity of a Google Street View-based audit. Int J Health Geogr 2014; 13(1):19.

94. Barrie H, Soebarto V, Lange J, Mc Corry-Breen F, Walker L. Using Citizen Science to Explore Neighbourhood Influences on Ageing Well: Pilot Project. Healthcare 2019; 7(4).

95. Griew P, Hillsdon M, Foster C, Coombes E, Jones A, Wilkinson P. Developing and testing a street audit tool using Google Street View to measure environmental supportiveness for physical activity. Int J Behav Nutr Phys Act 2013; 10(103).

96. Marco M, Gracia E, Martín-Fernández M, López-Quílez A. Validation of a Google Street View-Based Neighborhood Disorder Observational Scale. J Urban Health 2017; 94(2):190–8.

97. Edwards N, Hooper P, Trapp GS, Bull F, Boruff B, Giles-Corti B. Development of a Public Open Space Desktop Auditing Tool (POSDAT): A remote sensing approach. Applied Geography 2013; 38:22–30. Available from: URL: https://www.sciencedirect.com/science/article/pii/S0143622812001361.

98. Lafontaine SJV, Sawada M, Kristjansson E. A direct observation method for auditing large urban centers using stratified sampling, mobile GIS technology and virtual environments. Int J Health Geogr 2017; 16(1):6.

99. Buman MP, Winter SJ, Sheats JL, Hekler EB, Otten JJ, Grieco LA et al. The Stanford Healthy Neighborhood Discovery Tool: a computerized tool to assess active living environments. Am J Prev Med 2013; 44(4):e41-e47.

100. Hanibuchi T, Nakaya T, Inoue S. Virtual audits of streetscapes by crowdworkers. Health Place 2019; 59:102203. Available from: URL: https://www.sciencedirect.com/science/article/pii/S1353829219304897.

101. Steinmetz-Wood M, Velauthapillai K, O'Brien G, Ross NA. Assessing the micro-scale environment using Google Street View: the Virtual Systematic Tool for Evaluating Pedestrian Streetscapes (Virtual-STEPS). BMC Public Health 2019; 19(1):1246.

102. Blinkert B, Weaver E. Residential environment and types of childhood. Humanities and social sciences 2015; 3(5):159–68.

103. Ruppert P, Tran M-C, Schmidt JA. Walk-Audits in der Stadtplanung und Gesundheitsförderung. In: Niederberger M, Finne E, editors. Forschungsmethoden in der Gesundheitsförderung und Prävention. Wiesbaden: Springer Fachmedien; 2021. p. 651–83.

104. Wäsche H, Panter L, Mehl R, Rapp S. Sport und Bewegung in der Karlsruher Oststadt: Ergebnisbericht. Karlsruhe; 2019. KIT Scientific Working Papers 105.

105. Prüfliste für Fußgängerfreundlichkeit (Walkability Checkliste): Wie gehfreundlich ist Ihr Quartier?; 2015.

106. Spielplatz-Check; 2010 [cited 2023 Apr 19]. Available from: URL: https://www.dgk.de/fileadmin/user_upload/Gesundheit-pdf/spielplatzcheck.pdf.

107. Nordbø ECA, Nordh H, Raanaas RK, Aamodt G. Promoting activity participation and well-being among children and adolescents: a systematic review of neighborhood built-environment determinants. JBI Evid Synth 2020; 18(3):370–458.

108. Lambert A, Vlaar J, Herrington S, Brussoni M. What is the relationship between the neighbourhood built environment and time spent in outdoor play? A systematic review. International Journal of Environmental Research and Public Health 2019; 16(20):3840.

109. Timperio A, Reid J, Veitch J. Playability: built and social environment features that promote physical activity within children. Current obesity reports 2015; 4:460–76.

110. Larouche R. Built environment features that promote cycling in school-aged children. Current obesity reports 2015; 4:494–503.

111. D’Haese S, Vanwolleghem G, Hinckson E, Bourdeaudhuij I de, Deforche B, van Dyck D et al. Cross-continental comparison of the association between the physical environment and active transportation in children: a systematic review. International Journal of Behavioral Nutrition and Physical Activity 2015; 12(1):1–14.

112. McGrath LJ, Hopkins WG, Hinckson EA. Associations of objectively measured built-environment attributes with youth moderate–vigorous physical activity: a systematic review and meta-analysis. Sports Medicine 2015; 45:841–65.

113. Ding D, Sallis JF, Kerr J, Lee S, Rosenberg DE. Neighborhood environment and physical activity among youth: a review. American Journal of Preventive Medicine 2011; 41(4):442–55.

114. van Hecke L, Ghekiere A, Veitch J, van Dyck D, van Cauwenberg J, Clarys P et al. Public open space characteristics influencing adolescents’ use and physical activity: A systematic literature review of qualitative and quantitative studies. Health Place 2018; 51:158–73.

115. Ikeda E, Hinckson E, Witten K, Smith M. Associations of children's active school travel with perceptions of the physical environment and characteristics of the social environment: a systematic review. Health Place 2018; 54:118–31.

116. Marzi I, Demetriou Y, Reimers AK. Social and physical environmental correlates of independent mobility in children: a systematic review taking sex/gender differences into account. Int J Health Geogr 2018; 17(1):1–17.

117. McCormack GR, Shiell A. In search of causality: a systematic review of the relationship between the built environment and physical activity among adults. International Journal of Behavioral Nutrition and Physical Activity 2011; 8:1–11.

118. van Holle V, Deforche B, van Cauwenberg J, Goubert L, Maes L, van de Weghe N et al. Relationship between the physical environment and different domains of physical activity in European adults: a systematic review. BMC Public Health 2012; 12:807.

119. Wang L, Wen C. The relationship between the neighborhood built environment and active transportation among adults: A systematic literature review. Urban Science 2017; 1(3):29.

120. Carlin A, Perchoux C, Puggina A, Aleksovska K, Buck C, Burns C et al. A life course examination of the physical environmental determinants of physical activity behaviour: A "Determinants of Diet and Physical Activity" (DEDIPAC) umbrella systematic literature review. PLoS One 2017; 12(8):e0182083.

121. Bonaccorsi G, Manzi F, Del Riccio M, Setola N, Naldi E, Milani C et al. Impact of the Built Environment and the Neighborhood in Promoting the Physical Activity and the Healthy Aging in Older People: An Umbrella Review. Int J Environ Res Public Health 2020; 17(17).

122. Schulz M, Romppel M, Grande G. Built environment and health: a systematic review of studies in Germany. Journal of Public Health 2018; 40(1):8–15.

123. Yang Y, Wu X, Zhou P, Gou Z, Lu Y. Towards a cycling-friendly city: An updated review of the associations between built environment and cycling behaviors (2007–2017). Journal of Transport & Health 2019; 14:100613.

124. Kärmeniemi M, Lankila T, Ikäheimo T, Koivumaa-Honkanen H, Korpelainen R. The built environment as a determinant of physical activity: a systematic review of longitudinal studies and natural experiments. Ann Behav Med 2018; 52(3):239–51.
